# The component of the m^6^A writer complex VIRMA is implicated in aggressive tumor phenotype, DNA damage response and cisplatin resistance in germ cell tumors

**DOI:** 10.1186/s13046-021-02072-9

**Published:** 2021-08-25

**Authors:** Vera Miranda-Gonçalves, João Lobo, Catarina Guimarães-Teixeira, Daniela Barros-Silva, Rita Guimarães, Mariana Cantante, Isaac Braga, Joaquina Maurício, Christoph Oing, Friedemann Honecker, Daniel Nettersheim, Leendert H. J. Looijenga, Rui Henrique, Carmen Jerónimo

**Affiliations:** 1 Cancer Biology and Epigenetics Group, Research Center of IPO Porto (CI-IPOP) / RISE@CI-IPOP (Health Research Network), Portuguese Oncology Institute of Porto (IPO Porto) / Porto Comprehensive Cancer Center (Porto.CCC), R. Dr. António Bernardino de Almeida, 4200-072 Porto, Portugal; 2 Department of Pathology and Molecular Immunology, ICBAS – School of Medicine and Biomedical Sciences, University of Porto (ICBAS-UP), Rua Jorge Viterbo Ferreira 228, 4050-513 Porto, Portugal; 3 Department of Pathology, Portuguese Oncology Institute of Porto (IPOP), R. Dr. António Bernardino de Almeida, 4200-072 Porto, Portugal; 4 Princess Máxima Center for Pediatric Oncology, Heidelberglaan 25, 3584 CS Utrecht, The Netherlands; 5 Department of Urology, Portuguese Oncology Institute of Porto (IPOP), R. Dr. António Bernardino de Almeida, 4200-072 Porto, Portugal; 6 Department of Medical Oncology, Portuguese Oncology Institute of Porto (IPOP), R. Dr. António Bernardino de Almeida, 4200-072 Porto, Portugal; 7 Department of Oncology, Hematology and Bone Marrow Transplantation with Section of Pneumology, Mildred Scheel Cancer Career Center HaTriCs4, University Cancer Center Hamburg, University Medical Center Hamburg-Eppendorf, Martinistraße 52, 20246 Hamburg, Germany; 8 Tumour and Breast Center ZeTuP St. Gallen, Rorschacher Strasse 150, 9006 St. Gallen, Switzerland; 9 Department of Urology, Urological Research Lab, Translational UroOncology, University Hospital Düsseldorf, 40225 Düsseldorf, Germany

**Keywords:** N6-methyladenosine, VIRMA, Epitranscriptomics, Germ cell tumors, CRISPR/Cas9, RNA modifications, CAM, Cisplatin, DNA repair

## Abstract

**Background:**

Germ cell tumors (GCTs) are developmental cancers, tightly linked to embryogenesis and germ cell development. The recent and expanding field of RNA modifications is being increasingly implicated in such molecular events, as well as in tumor progression and resistance to therapy, but still rarely explored in GCTs. In this work, and as a follow-up of our recent study on this topic in TGCT tissue samples, we aim to investigate the role of N6-methyladenosine (m^6^A), the most abundant of such modifications in mRNA, in in vitro and in vivo models representative of such tumors.

**Methods:**

Four cell lines representative of GCTs (three testicular and one mediastinal), including an isogenic cisplatin resistant subline, were used. CRISPR/Cas9-mediated knockdown of VIRMA was established and the chorioallantoic membrane assay was used to study its phenotypic effect in vivo.

**Results:**

We demonstrated the differential expression of the various m^6^A writers, readers and erasers in GCT cell lines representative of the major classes of these tumors, seminomas and non-seminomas, and we evidenced changes occurring upon differentiation with all-trans retinoic acid treatment. We showed differential expression also among cells sensitive and resistant to cisplatin treatment, implicating these players in acquisition of cisplatin resistant phenotype. Knockdown of VIRMA led to disruption of the remaining methyltransferase complex and decrease in m^6^A abundance, as well as overall reduced tumor aggressiveness (with decreased cell viability, tumor cell proliferation, migration, and invasion) and increased sensitivity to cisplatin treatment, both in vitro and confirmed in vivo. Enhanced response to cisplatin after VIRMA knockdown was related to significant increase in DNA damage (with higher γH2AX and GADD45B levels) and downregulation of XLF and MRE11.

**Conclusions:**

VIRMA has an oncogenic role in GCTs confirming our previous tissue-based study and is further involved in response to cisplatin by interfering with DNA repair. These data contribute to our better understanding of the emergence of cisplatin resistance in GCTs and support recent attempts to therapeutically target elements of the m^6^A writer complex.

**Supplementary Information:**

The online version contains supplementary material available at 10.1186/s13046-021-02072-9.

## Background

RNA modifications have been actively explored in the last decade (with more than 140 such modifications uncovered so far [1, 2]). They are involved in several biological processes, including differentiation, metabolism, embryogenesis and immune response; also, compelling evidence has implicated such modifications (which may be observed in several RNA molecules, including mRNA and non-coding RNAs) in cancer development [3, 4]. The most abundant of these modifications is N6-methyladenosine (m^6^A), which is introduced/removed by proteins called “writers” and “erasers”, respectively, and then bound to “readers”, which target RNAs for their ultimate destination [5, 6]. Deregulation of such players has been demonstrated to determine various aspects of tumorigenesis, with implications in prognosis and patient outcome, across tumor models [7, 8]. The field is expanding and, recently, research has been directed towards targeting these alterations therapeutically [9]. However, still few works have focused on testicular germ cell tumors (TGCTs) [10–14].

TGCTs are the most common malignancies affecting male individuals aged 15–39 years, worldwide. The most common TGCTs are derived from germ cell neoplasia in situ (GCNIS) and comprise seminoma (SE) and non-seminoma (NS) subtypes, the latter including embryonal carcinoma (EC), choriocarcinoma (CH), yolk sac tumor (YST) and teratoma (TE) [15]. This tumor model is highly associated with developmental biology and phenomena related to pluripotency and differentiation [16, 17], in which m^6^A is implicated [18–21], raising the hypothesis that m^6^A is also important for the biology of these tumors. Indeed, the various m^6^A-related proteins have been showed to be differentially expressed in TGCT subtypes (serving as biomarkers of the disease [10, 11]). Additionally, more recently, the m^6^A writer METTL3 has been implicated in cisplatin resistance, specifically in the seminoma-like cell line TCam-2 [13], supporting that m^6^A regulation of gene expression may contribute to an aggressive phenotype.

In a previous work, we have proven the relevance of VIRMA (also known as KIAA1429) in these tumors [10]. VIRMA (or vir-like m^6^A methyltransferase associated) is a critical part of the methyltransferase complex (which includes METTL3, the catalytic component, as well as other players such as WTAP and METTL14 that work together to stabilize and allow the functionality of the complex) and was shown to contribute to cancer progression in multiple malignancies by regulating cell cycle progression, migration, invasion, resistance to apoptosis and tumor growth, in both m^6^A dependent and independent manners [22]. In our cohort of 122 TGCT patients, VIRMA (and the m^6^A reader YTHDF3) was found to be significantly upregulated (at both transcript and protein levels) in SE compared to NS subtypes, confirming in silico data reporting these two m^6^A-related players as the most upregulated in TGCTs. Importantly, expression of both players was positively correlated and associated with m^6^A abundance, suggesting that this writer/reader pair cooperates to introduce m^6^A modification in TGCTs, in a manner dependent on histological subtype. We also found VIRMA to be highly expressed in cisplatin-exposed metastatic tumor samples, suggesting a role in tumor aggressiveness and in cisplatin response, requiring further investigation. Therefore, following these previous tissue-based observations [10], we aimed herein to explore in more detail the expression patterns of the several m^6^A-related players related to differentiation and to cisplatin resistance. We demonstrate that VIRMA contributes to NCCIT cells tumor aggressiveness and to cisplatin resistance, both in vitro and in vivo*,* by regulating DNA damage response.

## Methods

### Cell lines and treatments

The (T)GCT cell lines TCam-2, NCCIT, 2102Ep and NT2 were kindly provided by Prof. Leendert Looijenga, and cultured as described [23]. Cell lines have been previously authenticated [details reported in [24]]. The cisplatin-resistant and cisplatin-sensitive clones of NCCIT were kindly provided by Prof. Daniel Nettersheim, and established by Dr. Christoph Oing and Prof. Friedemann Honecker. The resistant isogenic subline (NCCIT-R) was derived from the parental cisplatin-sensitive cell line (NCCIT-P) through repeated exposure to increasing sub-lethal doses of cisplatin, as reported previously [25].

To evaluate cells differentiation effects on m^6^A-related players, the NS cell lines NCCIT, NT2 and 2102Ep were treated with the differentiation-inducing agent all-trans retinoic acid - ATRA (STEMCELL™ Technologies). Briefly, cells were seeded on T25 culture flasks, left to adhere for 24 h and then treated with 10 μM of the drug for 10 days, with drug renewal every 2 days. Importantly, cell morphology analysis in NT2 cell line, as well as assessment of pluripotency/differentiation markers, were performed to document neuronal-like differentiation of the cells, as previously reported by our team [24].

Cells were treated with cisplatin (provided by IPO Porto’s Department of Pharmacy) as described in [26] (see below for details).

### RNA extraction, cDNA synthesis and real-time quantitative polymerase chain reaction (RT-qPCR)

Total RNA was extracted from cell lines using TRIzol (Invitrogen). RNA quantification and purity were assessed with NanoDrop™ Lite Spectophotometer (Cat. ND-LITE, Thermo Scientific™). cDNA synthesis (1000 ng) was performed as described earlier [10]. RT-qPCR was run in LightCycler® 480 multiwell plate system (Product no. 05015243001, Roche) using commercially available gene expression assays and primers (Supplementary Table 1). For normalization purposes, *18S rRNA* and *GUSB* were used as housekeeping genes. cDNA obtained from Human Reference Total RNA (Cat. 750,500, Agilent Technologies®) was used as positive control and two non-template controls were included in each plate. Data was plotted using the 2^^-ΔΔCt^ method. Five biological replicates were used, and reactions were also performed in triplicate.

### Western blot

Total protein was extracted from cells, in biological triplicates, using the radioimmunoprecipitation assay buffer (Santa Cruz Biotechnology Inc., USA) complemented with protein inhibitor cocktail (Roche). After 15 min on ice, samples were centrifuged at 13,000 rpm for 30 min at 4 °C and the supernatant was collected. Protein was quantified using a Pierce BCA Protein Assay Kit (Thermo Scientific Inc., USA), according to the manufacturer’s instructions. Aliquots of 30 μg total protein from each cell line were resuspended in loading buffer, denatured at 95 °C for 5 min, and loaded in 8% or 10% polyacrylamide gels (as appropriate), where they were separated by size through sodium dodecyl sulphate-polyacrylamide gel electrophoresis at 120 V. Then, proteins were transferred to 0.2 μm nitrocellulose membranes (Bio-Rad Laboratories Inc., Hercules, CA, USA) using 25 mM Tris-base/glycine buffer and a Trans-Blot Turbo Transfer system (Bio-Rad) at 25 V and 1.3 mA for 10–15 min, as appropriate. Membranes were blocked with 5% bovine serum albumin (BSA; Santa Cruz, USA) or 5% dry milk in TBS with 0.1% Tween (pH = 7.6) as appropriate, and then incubated with the respective primary antibodies (Supplementary Table 2). Lastly, membranes were incubated with secondary antibody coupled with horseradish peroxidase (Cell Signaling), for 1 h at room temperature. Quantification was performed by band densitometry analysis using the ImageJ software (version 1.6.1, National Institutes of Health), by comparing the specific protein band intensity with the loading control beta-actin. All quantifications were done in triplicates, reported as mean ± SD.

### Immunofluorescence

Cells were fixed with 4% paraformaldehyde, permeabilized with 0.25% Triton-X and blocked with 5% BSA at room temperature. Cells were incubated with anti-Phospho Histone γH2AX primary antibody (1:500) and then with secondary antibody (1:1000) anti-rabbit immunoglobulin G (Alexa Fluor™ 488 goat, A11008, Invitrogen, Carlsbad, CA, USA). Images were captured with the fluorescence microscope Olympus IX51 (400× magnification) with digital camera Olympus XM10, using the CellSens software. Quantification was performed in Image J, by measuring signal intensity and normalizing to the number of cells assessed.

### m^6^A quantification using ELISA and dot blot

The m^6^A RNA methylation quantification kit (ab185912; Abcam, Cambridge, United Kingdom) was used to measure the m^6^A content of total RNA, following the manufacturer’s instructions. Positive and negative controls provided with the kit were used to compute m^6^A levels, according to the manufacturer’s instructions. This assessment was complemented by m^6^A dot blot assay. Briefly, 2000 ng of RNA were denatured at 95 °C for 3 min and then pipetted into nitrocellulose membranes. Membranes were dryed for 30 min at 37 °C following UV light exposure (125mJoules/cm^2^) for allowing crosslink between RNA and the membrane. Then, membranes were washed in TBS-0.1% Tween, blocked in 5% dry milk and incubated with primary antibody for m^6^A (Abcam, 1:1000) at 4 °C, overnight. After that, membranes were incubated with anti-rabbit secondary horseradish peroxidase conjugated antibody (1:5000, Cell Signaling Technology) for 1 h at room temperature and the signal was detected by chemiluminescence. Methylene blue staining solution (0.02% methylene blue in 0.3 M sodium acetate, pH 5.5) was used as loading control.

### Prediction of m^6^A modification sites

The publicly available sequence-based RNA adenosine methylation site predictor (SRAMP, http://www.cuilab.cn/sramp [27]) was used to analyze m^6^A modification in specific targets. FASTA sequence was introduced for each target and the corresponding score and confidence on m^6^A deposition per site was annotated.

### Patient samples and immunohistochemistry

Tumor samples were retrospectively selected from type II TGCT patients undergoing radical inguinal orchiectomy between 2005 and 2018 at Portuguese Oncology Institute of Porto (IPO Porto). Samples of a total of 96 TGCT patients were included (Supplementary Table 3); distinct tumor components within mixed tumors were assessed individually, resulting in a total of 147 tumor samples analyzed. Additionally, 11 samples of post-chemotherapy residual masses (eight residual mature teratomas and three cisplatin-resistant non-teratoma tumor samples) were included. Specimens were routinely fixed in formalin and embedded in paraffin and a representative tumor block, with > 70% tumor cellularity and low necrosis content was selected for immunohistochemical evaluation. All histological material was reviewed by the same TGCT-expert histopathologist according to the most recent 2016 World Health Organization classification (full cohort characteristics reported in [15]). Patients were staged according to the most recent American Joint Committee on Cancer 8th edition. Follow-up was last updated in May 2020. This study was approved by IPO Porto’s Ethics Committee (CES-IPO-1/018).

Three-μm thick sections were deparaffinized and submitted to antigenic recovery (20 min with EDTA buffer pH = 8 in microwave). The immunohistochemistry protocol used is described in detail in [10]. Slides were incubated for 1 h with anti-METTL3 primary antibody (1:750, monoclonal [EPR18810], abcam [ab195352]), at room temperature. Tissue of lung adenocarcinoma was used as external positive controls in each run. Negative controls consisting in omission of primary antibodies were included per run. Both percentage of stained cells (in 25% intervals) and intensity of staining (considered as “weak”, “moderate” and “strong”, as defined in [28]) were ascertained, and results were combined in a “Combined Score (CS)” (percentage x intensity).

### CRIPSR/Cas9-mediated knockdown of VIRMA

NCCIT cell line (the one showing the highest resistance to cisplatin compared to 2102Ep and NT2, as documented in our previous study [26]) was chosen to perform VIRMA knockdown by plasmids carrying the CRISPR/Cas9 system containing a guide RNA sequence (available in [29]) targeting this gene (obtained from GenScript, Piscataway, NJ). The representativity of the sequence (targeting the two available protein coding transcripts of VIRMA) was previously validated by our group [29]. Briefly, for plasmid transfection, Lipofectamine® 3000 reagent (Invitrogen, USA) was used according to manufacturers’ instructions, followed by selection of cells which incorporated the CRISPR/Cas9 system with puromycin. After selection, cells were expanded, total protein was extracted to confirm VIRMA protein downregulation (at the beginning, and midway through experiments and before the in vivo assay) and phenotypic assays were performed. Wild-type cells were used as controls, as well as cells transfected with a scrambled (non-specific guide) vector, used as controls for all experiments (to control for off-target effects).

### Cell viability assays

Briefly, the viability assay was performed for NCCIT scramble and VIRMA knockdown cells at 24 h, 48 h and 72 h. A second experiment consisted of exposure of cells to 1 μM, 3.3 μM and 10 μM cisplatin for 72 h (as performed in [26]) after which viability was assessed. Cells were plated into 96-well plates in medium at density of 6000 cells/well (seeding density previously optimized) and incubated overnight, at 37 °C in 5% CO_2_.

For the viability assay, Resazurin (Canvax Biotech, Córdoba, Spain) was used. The culture medium was removed, and cells were incubated for 3 h at 37 °C with 100 μL of 1:10 Resazurin solution in culture medium. The solution was then removed, and spectrophotometric measurement was done at 560 nm (reference wavelength: 600 nm) in a microplate reader (Fluostar Omega, BMG Labtech, Germany). Wells with the Resazurin solution were used as blank to correct the OD values. ODs obtained for each time point were all normalized for the 0 h-time point. Results were normalized to the scramble condition. All experiments were performed in biological triplicates, each with experimental triplicates.

### Proliferation assays

The BrdU assay was performed for scramble and VIRMA knockdown cells at 24 h, 48 h and 72 h. Cells were plated into 96-well plates in medium at density of 6000 cells/well (seeding density previously optimized) and incubated overnight, at 37 °C in 5% CO_2_. At each timepoint, cells were previously incubated with 20 μM BrdU labelling solution for 12 h. After removing labelling medium, cells were fixed for 30 min at room temperature with FixDenat solution, whereafter which anti-BrdU-POD antibody (1:100) was added. After 90 min the antibody was removed, and cells were rinsed 3 times with 1x PBS. The immune complex formed was detected by adding 100 μl/well of substrate solution and incubated for 5–10 min, until color development. Then, the reaction was stopped with 1 M H_2_SO_4_ added to each well, and the reaction product was quantified in a microplate reader by measuring absorbance at 450 nm (reference wavelength: 690 nm). ODs obtained for each time point were normalized for the 0 h-time point. Results were normalized to the scramble condition. All experiments were performed in biological triplicates, each in experimental triplicates.

### Invasion and migration assays

Migration and invasion capacities were assessed for scramble and VIRMA knockdown cells using polycarbonate insert chambers (Thermo Fisher Scientific) and BD BioCoat Matrigel Invasion Chambers (BD Biosciences), respectively. After rehydration of inserts in DMEM medium for 2 h at 37 °C, cells were seeded at density of 5 × 10^4^ cells/insert and incubated 24 h at 37 °C in 5% CO_2_. Then, non-migrating/non-invading cells were removed by swab and migrated/invaded cells were fixed with 4% PFA for 2 min and with cold methanol during 20 min, followed by cell staining with Cristal Violet for 10 min. Membranes were photographed in Olympus SZx16 stereomicroscope (16x), and migrating/invading cells were counted using the Image J software (version 1.41; National Institutes of Health). At least three independent experiments were performed.

### Chorioallantoic membrane (CAM) assay

Thirty-six fresh fertilized eggs (PintoBar, Lda, Portugal) were incubated at 37 °C in a humid environment. After 3 days of embryonic development, a window was opened into the eggshell under aseptic conditions. On day 9, NCCIT scramble and VIRMA knockdown cells suspensions in growth factor-reduced Matrigel (BD Biosciences) were seeded on the CAM. Then, on day 14, a treated group (*n* = 8 for scramble condition and *n* = 9 for VIRMA knockdown condition), randomly selected, received 3.3 μM cisplatin whereas the remaining control groups received only 1% PBS in complete DMEM. Lastly, on day 17, tumors were dissected and included in a paraffin block. Microtumor images were obtained on day 14 (0 h of treatment) and at day 17 (72 h of treatment). Relative size and blood vessel counting in *in ovo* condition was assessed using CellSens software (version V0116, Olympus). *Ex ovo* pictures were also obtained. H&E slides were scanned in a Ventana DP200 Slide Scanner (Roche) and scored by a TGCT-expert histopathologist, including tumor size, number of micro-vessels at the tumor periphery, presence of individual tumor cells infiltrating vessels and estimation of viability/response to cisplatin treatment.

### Statistical analysis

Data was tabulated using Microsoft Excel 2016 and analyzed and plotted using GraphPad Prism 6 and SPSS v27. Percentages were calculated based on the number of cases with available data. Non-parametric tests (Mann-Whitney U and Kruskal-Wallis tests) were used for comparing continuous variables among all samples (patients and cell lines), as necessary. All *p*-values were adjusted for multiple comparisons (Dunn’s test and Bonferroni correction, as appropriate). Chi-square and Fisher’s exact test were used as necessary for establishing associations between categorical variables. Statistical significance was set at *p* < 0.05 and is reported in graphs as following: * p < 0.05; ** *p* < 0.01; *** *p* < 0.001; **** *p* < 0.0001.

## Results

### m^6^A and its writers, readers and erasers are differentially expressed among (T) GCT cell lines, and their expression is altered upon differentiation

**Fig. 1:**
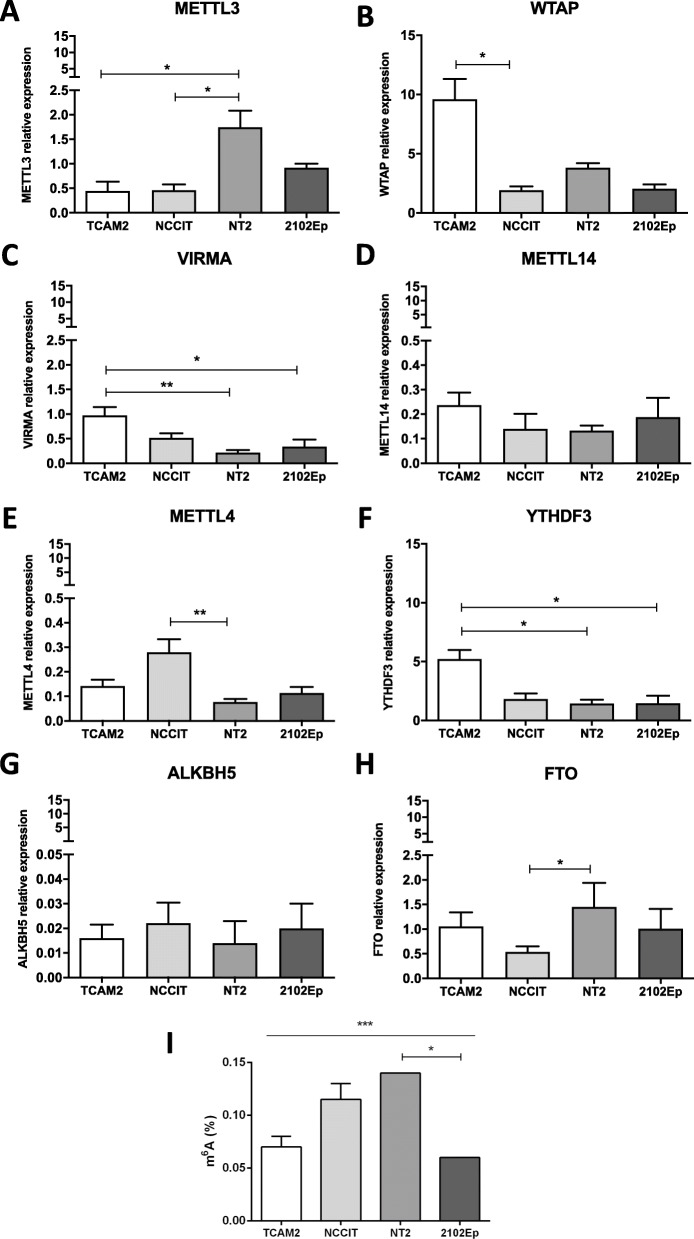
m^6^A abundance and expression of related writers, readers and erasers in TGCT cell lines. **A**-**H**: Relative mRNA expression levels of METTL3, WTAP, VIRMA, METTL14, METTL4, YTHDF3, ALKBH5 and FTO in four TGCT cell lines. Results are normalized to GUSB/18S rRNA, and plotted in 2^^-ΔΔCt^ format; I: Percentage of m^6^A abundance detected by specific ELISA method in four TGCT cell lines. * *p* < 0.05; ** *p* < 0.01; *** *p* < 0.001

First, we aimed to characterize mRNA expression of the several m^6^A-related players in the four GCT cell lines. TCam-2 is a SE-like cell line, while NCCIT, NT2 and 2102Ep are representative of NS, specifically containing EC and with distinct p53 status (further description available in [30]). Specifically, writer VIRMA and reader YTHDF3 were significantly upregulated in SE-like TCam-2 cells, reflecting our previously published results in primary tumor samples, where both players were upregulated in SE as well [10]. Additionally, WTAP was also expressed at significantly higher levels in TCam-2. On the contrary, METTL3 was significantly higher expressed in NT2 cells, and METTL4 in NCCIT cells, with no significant differences observed for METTL14. Overall, the highest transcript levels were found for WTAP and YTHDF3, followed by METTL3 and VIRMA. Among the erasers, ALKBH5 was expressed at a very low range, with FTO being the most expressed, and significantly upregulated in NT2 cells (Fig. 1A-H). Finally, m^6^A amount (which varied between 0.04 and 0.14%, similar to the range detected in another publication [11]) was higher in NCCIT and NT2 cell lines compared to 2102Ep and TCam-2 cells (Fig. 1I). These results support that m^6^A is indeed detected in all four representative cell lines, but also highlights cells’ variability in m^6^A abundance and in expression levels of m^6^A-related players, which may be due to their distinct molecular background and developmental potential (in consonance with our findings in primary tumors, with differential expression abundance of m^6^A according to histology and differentiation [10]).

As mentioned, m^6^A is overall strongly implicated in cell fate decisions and regulation of pluripotency [18–21]. Since our hypothesis was that m^6^A would also be relevant in tumors’ degree of differentiation, we assessed the impact of ATRA-induced differentiation (a classical feature of ATRA treatment in these cell lines, as described in [24, 31]) on the total amount of m^6^A and on the expression levels of its key effectors, comparing with the parental non-differentiated matched cells. We found a statistically significant downregulation of METTL3, WTAP, VIRMA, METTL14, METTL4 and YTHDF3 in ATRA-treated NCCIT cells compared to the vehicle (Supplementary Fig. 1A). Expression of eraser ALKBH5 was higher in differentiated NCCIT cells, although it did not reach statistical significance. A similar pattern was observed for NT2 cells, with significant downregulation of METTL3, VIRMA, METTL4 and YTHDF3, in this instance accompanied by significant upregulation of both erasers ALKBH5 and FTO (Supplementary Fig. 1B). Similarly, for 2102Ep cell line, a significant downregulation of WTAP, METTL14, METTL4 and YTHDF3 was found, but also with FTO downregulation (Supplementary Fig. 1C). Overall, these data indicate that the expression levels of several writers (and of reader YTHDF3) are significantly reduced upon ATRA exposure and support the involvement of m^6^A in the differentiation process. Indeed, and importantly, the amount of m^6^A in ATRA-differentiated cells was significantly reduced in all three cell lines (Supplementary Fig. 1D).

### m^6^A-related players are differentially expressed in cisplatin-resistant and cisplatin-sensitive NCCIT cells

**Fig. 2:**
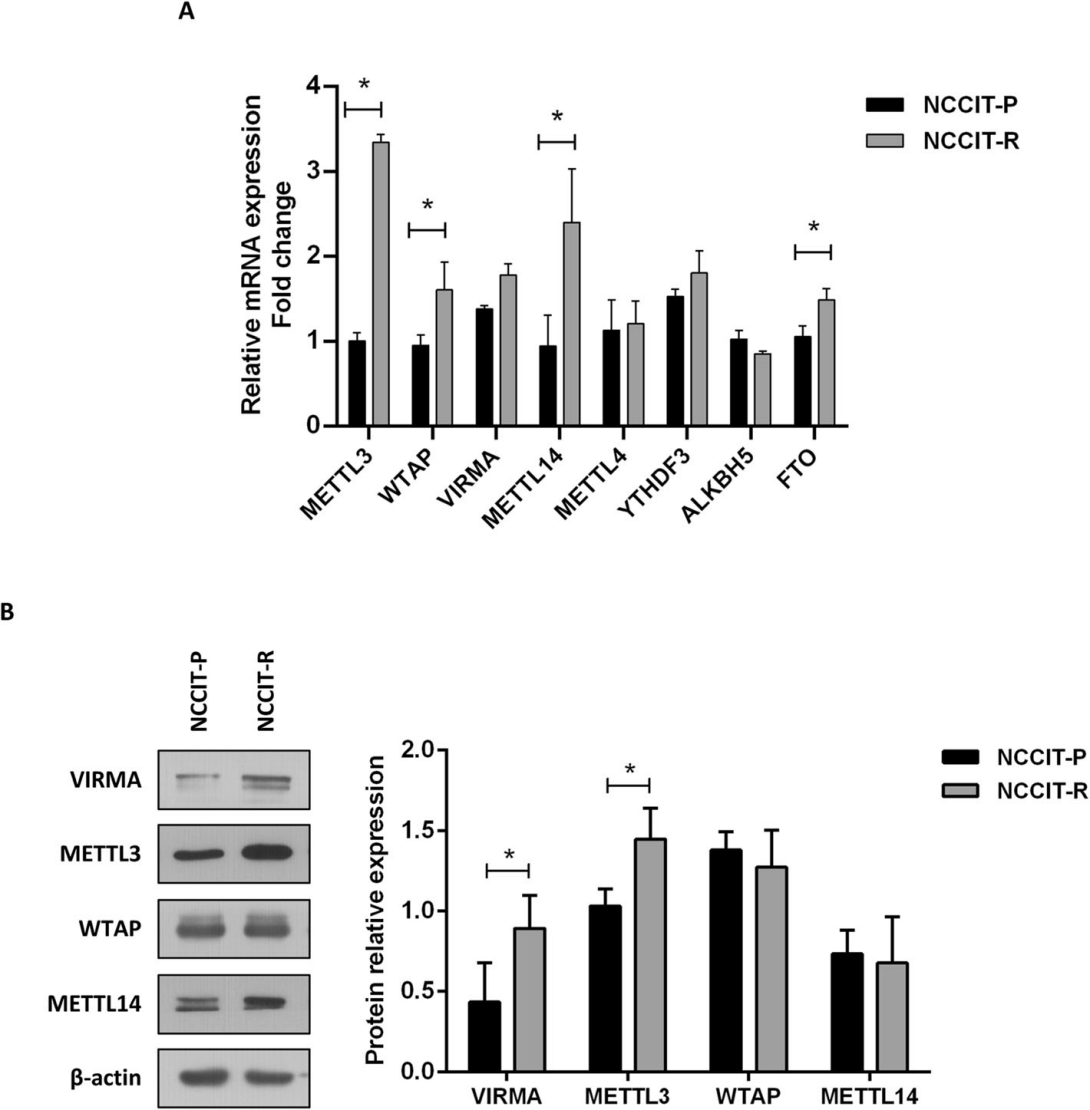
Differential expression of m^6^A related players among NCCIT parental and resistant cell line clones. **A**: Relative mRNA expression levels of METTL3, WTAP, VIRMA, METTL14, METTL4, YTHDF3, ALKBH5 and FTO. Results are normalized to GUSB/18S rRNA, computed in 2^^-ΔΔCt^ format and expressed as fold-change compared to NCCIT-P levels; **B**: Relative protein expression levels of METTL3, VIRMA, WTAP and METTL14. Results are normalized to ß-actin and expressed as fold-change compared to NCCIT-P levels. * *p* < 0.05

We then compared mRNA expression of selected players among NCCIT matched parental and cisplatin resistant clones (chosen for being the pair with highest documented differential sensitivity to cisplatin in our previous characterization [26]). METTL3 (the catalytic member of the writer complex) transcript was significantly more expressed in NCCIT-R compared to NCCIT-P (approximately 3-fold change), corroborating a previous report for TCam-2 cells [13]. Overall, the remainder members of the m^6^A writer complex as well as YTHDF3 reader showed increased expression in NCCIT-R compared to NCCIT-P, achieving significance for WTAP and METTL14 (Fig. 2A). At protein level, significantly higher expression of VIRMA and METTL3 was also observed compared to NCCIT-P (Fig. 2B).

Considering the recent report implicating METTL3 in cisplatin resistance [13], we assessed its expression in patient tissue samples. METTL3 immunoexpression in our TGCT patient cohort was significantly higher in NSs compared to SEs (Supplementary Fig. 2A), in line with protein expression found in our cell line models (see above). When discriminating the various histologies, SEs were the tumor components disclosing the lowest expression of this protein compared to either EC, YST, CH or TE (Supplementary Fig. 2B). Importantly, of the 11 post-chemotherapy residual masses, 9 (81.8%) depicted high immunoexpression score for METTL3 (Supplementary Fig. 2C). Although a larger proportion of stage II/III cases showed higher immunoexpression scores compared to stage I patients, it did not achieve statistical significance (Supplementary Fig. 2D). Also, although patients with high METTL3 immunoexpression showed shorter relapse-free survival, no statistical significance was attained, likely due to the limited number of relapse events (Supplementary Fig. 2E). Illustrative images of METTL3 immunoexpression are represented in Supplementary Fig. 3.

### VIRMA contributes to tumor cell aggressiveness and to cisplatin resistant phenotype in vitro

**Fig. 3:**
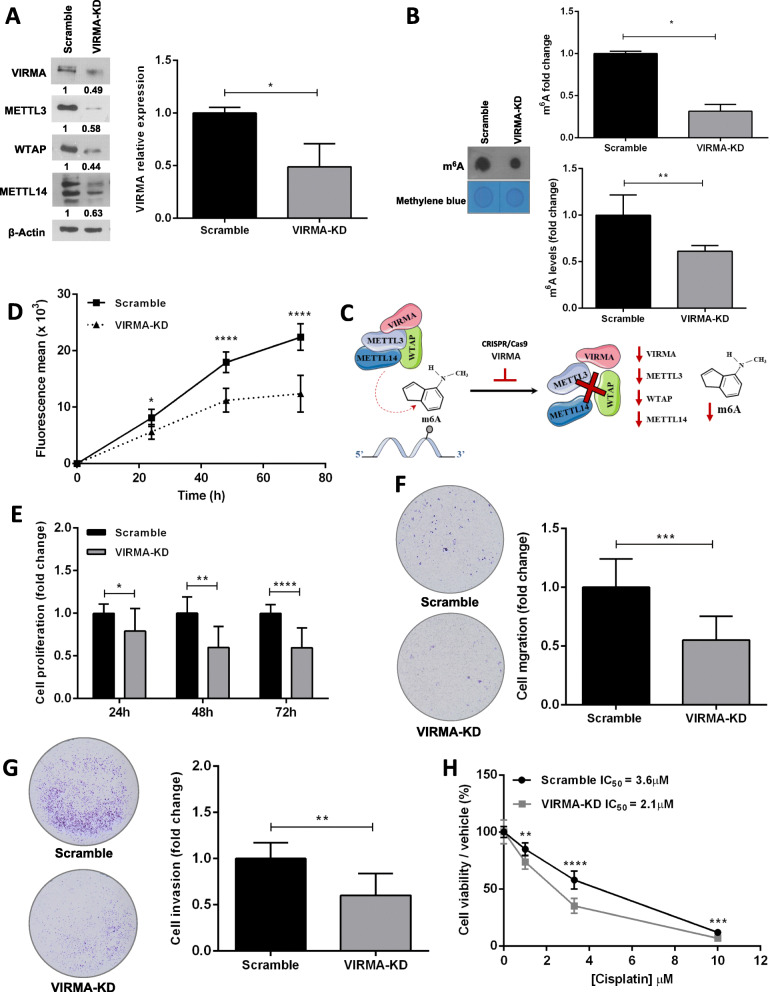
Knockdown of VIRMA attenuates the malignant phenotype and enhances sensitivity to cisplatin in vitro. **A**: CRISPR/Cas9-mediated knockdown of VIRMA in NCCIT cells (~ 50% reduction), leading to decreased protein expression of other members of the writer complex – METTL3, WTAP and METTL14. Results are normalized to ß-actin and expressed as fold-change compared to scramble condition; **B**: Relative levels of m^6^A, expressed as fold-change compared to scramble condition, both by ELISA kit (top) and dot blot (bottom, normalized to methylene blue); **C** – Illustration of the m^6^A writer complex and hypothesis related to its disruption upon VIRMA knockdown; **D** – Tumor cell growth curves in VIRMA knockdown cells compared to scramble condition along 72 h; **E** – Measurement of tumor cell proliferation by BrdU assay along 72 h. Results are expressed as fold-change compared to scramble condition; **F** – Measurement of migration capacity. Results are expressed as fold-change compared to scramble condition; **G** - Measurement of invasion capacity. Results are expressed as fold-change compared to scramble condition; **H** – Cell viability curves for NCCIT-VIRMA knockdown and scramble cells treated with cisplatin. Results are expressed as percentage cells surviving, normalized to the vehicle. IC_50_ concentration is indicated for each condition. * *p* < 0.05; ** *p* < 0.01; **** *p* < 0.0001

Following our previous in silico analysis and also our work in patient samples [3, 10] describing VIRMA as a relevant biomarker of TGCTs (as well as for urological neoplasms in general), particularly strong VIRMA immunoexpression was depicted by 14/14 cisplatin-exposed resistant patient samples. This way we performed CRISPR/Cas9-mediated knockdown of this component of the methyltransferase complex in NCCIT cells. We confirmed VIRMA knockdown (~ 50%) compared to scramble cells, which also resulted in reduced protein expression of the remaining components of the methyltransferase complex, including METTL3, WTAP and METTL14. Importantly, a significant m^6^A decrease was observed in VIRMA knockdown cells, both by ELISA and dot blot assays (Fig. 3A-B). These results support that VIRMA-knockdown leads to efficient functional disruption of the m^6^A writer complex (Fig. 3C).

Moreover, NCCIT-VIRMA knockdown cells displayed a significant decrease in cell growth compared to controls (Fig. 3D). This was further confirmed by showing a significant decrease in tumor cell proliferation in NCCIT-VIRMA knockdown cells compared to controls, ascertained by BrdU incorporation (Fig. 3E). Additionally, VIRMA knockdown cells exhibited significantly less invasion as well as migration ability compared to scramble cells (Fig. 3F-G).

Finally, and because these data support an oncogenic role of VIRMA, we hypothesized whether it could also be implicated in regulating response to cisplatin treatment. In fact, NCCIT-VIRMA knockdown cells showed significantly reduced viability after cisplatin exposure compared to control cells (with IC_50_ values for cisplatin decreasing from 3.6 μM in scramble cells to 2.1 μM in VIRMA knockdown, corresponding to a 1.7x decrease, Fig. 3H). This differential sensitivity to cisplatin was not attributed to cell differentiation, as no significant changes in expression of NANOG, OCT3/4 or SOX2 between knockdown and scramble cells were found, nor morphological changes, as documented in [24] (Supplementary Fig. 4).

### VIRMA modulates response to cisplatin by interfering with DNA damage response

**Fig. 4:**
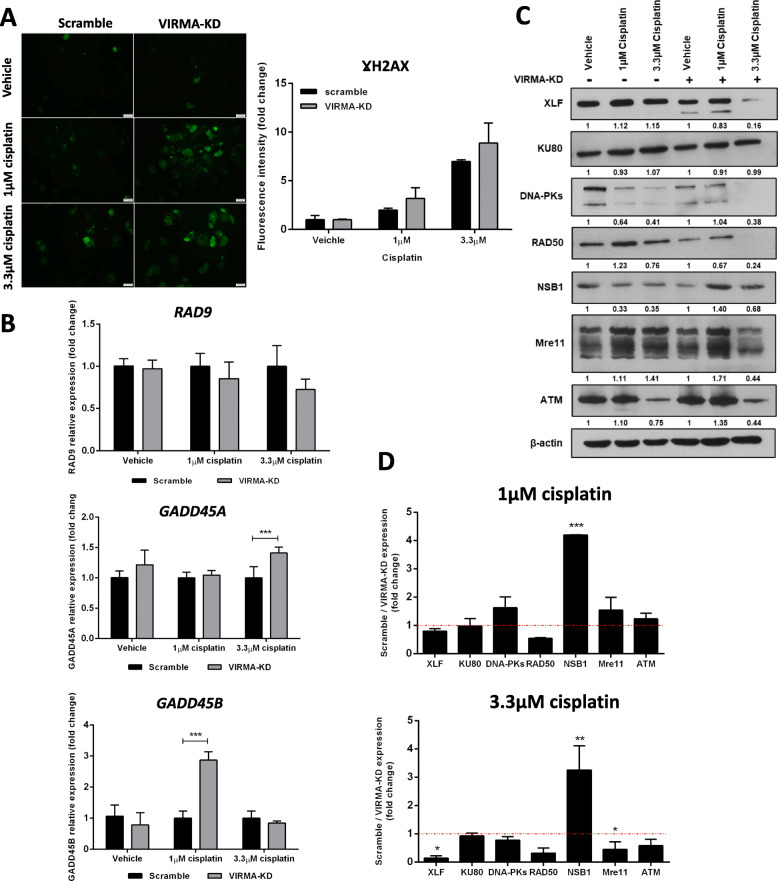
Knockdown of VIRMA contributes cisplatin sensitivity by impairing DNA repair. **A** - γH2AX levels in VIRMA knockdown and scramble conditions upon treatment with 1 μM and 3.3 μM cisplatin. Results are computed as fluorescence intensity, normalized to number of cells, and expressed as fold-change; **B** – Transcript levels of RAD9, GADD45A and GADD45B in VIRMA knockdown and scramble conditions upon treatment with 1 μM and 3.3 μM cisplatin. Results are normalized to GUSB, computed in 2^^-ΔΔCt^ format and expressed as fold-change in VIRMA knockdown compared to scramble condition; **C** – Protein expression of players involved in homologous recombination and non-homologous end joining DNA repair pathways in VIRMA knockdown and scramble conditions upon treatment with 1 μM and 3.3 μM cisplatin. Results are normalized to ß-actin. Values below each band refer to densitometry as fold-change compared to vehicle in the two independent group conditions (scramble and VIRMA knockdown); **D** – Related to the blots presented in C, the graphs show plotted the fold-change expression of VIRMA knockdown cells compared to scramble condition, for each concentration. *** *p* < 0.001

NCCIT-VIRMA knockdown cells exposed to 1 μM and 3.3 μM cisplatin showed higher γH2AX levels compared to scramble cells (Fig. 4A). Moreover, VIRMA knockdown cells displayed significantly higher GADD45A and GADD45B expression than scramble-treated cells exposed to 3.3 μM and 1 μM cisplatin, respectively, although no significant changes were found for RAD9 (Fig. 4B). These data indicate an enhanced DNA damage induction upon cisplatin exposure in VIRMA knockdown cells.

Since DNA double-strand breaks are understood to be the most damaging results of cisplatin-DNA-adduct formation, we explored targets of the homologous and non-homologous DNA repair pathways in our cell line models. Interestingly, VIRMA knockdown cells showed a significantly more prominent decrease of XLF and MRE11 protein expression compared to the respective scramble condition upon cisplatin exposure. No significant changes in ATM (a central regulator/transducer of the double-strand break repair via the homologous recombination pathway) expression were found (Fig. 4C-D). Finally, SRAMP analysis confirmed that both XLF and MRE11 displayed several predicted m^6^A sites with “high confidence” (*n* = 4 for XLF and *n* = 2 for MRE11) and also with “very high confidence” for XLF (*n* = 1, combined score 0.904, Supplementary Table 4), the most differentially expressed gene in VIRMA knockdown cells exposed to cisplatin. These results further indicate that the observed alterations in gene expression are occurring in a m^6^A-dependent way.

### VIRMA contributes to tumor cell aggressiveness and to cisplatin resistant phenotype in vivo

**Fig. 5:**
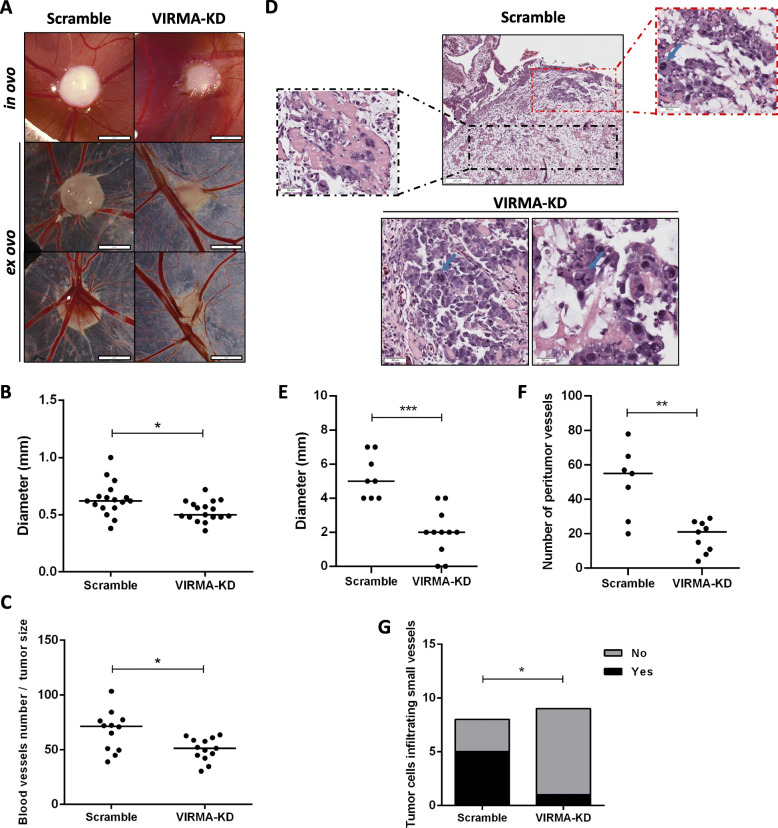
Knockdown of VIRMA attenuates the malignant phenotype in vivo. **A** – Macroscopic view of tumor formation (*in ovo* and *ex ovo*) and neo-angiogenesis in NCCIT scramble and VIRMA knockdown experimental conditions. Notice reduced size and decreased number of vessels in VIRMA knockdown tumors; **B** and **C** – Distribution of macroscopic tumor size and number of peri-tumor vessels (normalized to size of tumor) in scramble and VIRMA knockdown conditions; **D** – Histological aspects of scramble and VIRMA knockdown tumors. Notice the highly vascularized tissue around the scramble tumor, and infiltration of individual clusters of tumor cells around and into blood vessels (black box inset). Notice the cellular atypia (red box inset), including mitotic figure (blue arrow), in a more cellular area. The two VIRMA knockdown tumors with higher tumor cellularity are also shown (notice the clear cellular atypia, with prominent nucleoli and mitotic figures – blue arrows – including an atypical tripolar mitosis); **E** and **F** - Distribution of tumor size and number of peri-tumor vessels assessed histologically in scramble and VIRMA knockdown conditions; **G** – Presence of images of invasion into blood vessels. * *p* < 0.05; ** *p* < 0.01; *** *p* < 0.001

Using the CAM assay, we demonstrated that VIRMA-knockdown tumors were significantly smaller (assessed macroscopically and histologically) compared to scramble condition, and in two out of eleven VIRMA knockdown inoculations no tumor was apparent at all. Moreover, VIRMA-knockdown tumors disclosed significantly lower number of vessels at tumor periphery, both macroscopically and histologically, even when normalizing for tumor size. Importantly, images of individual clusters of cells infiltrating small vessels were more impressive in the scramble tumors than in VIRMA-knockdown tumors, which depicted lymphatic vessel invasions in only 1/9 tumors (Fig. 5).

**Fig. 6:**
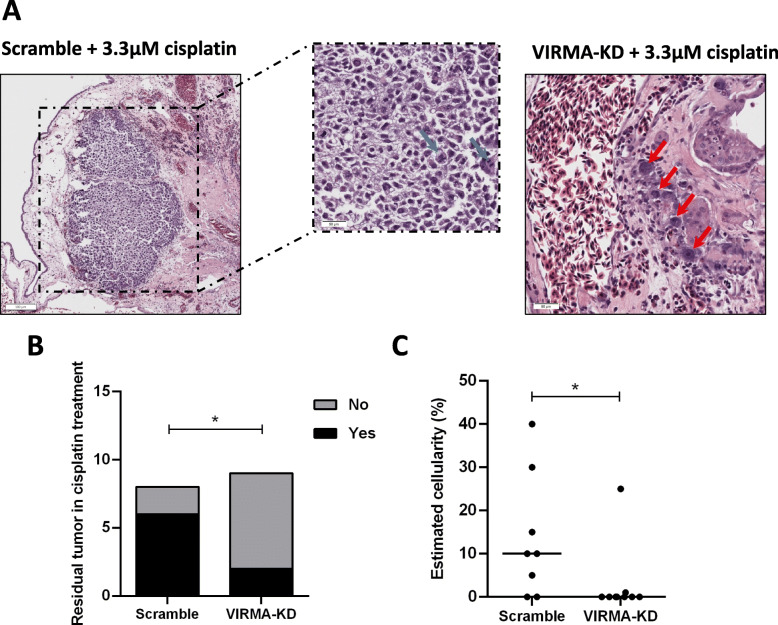
Knockdown of VIRMA enhances sensitivity to cisplatin in vivo. **A** – Histological view of residual tumors after cisplatin treatment in both scramble and VIRMA knockdown conditions. Notice the higher residual cellularity in scramble conditions, with clear atypia, mitotic figures (blue arrows) and irregular nuclear contour (black box inset). Compare with one of the two VIRMA knockdown tumors showing residual viable tumor cells after cisplatin, where only a very small cluster of individual cells remained across the whole sections (red arrows); **B** and **C** – Presence of residual viable tumor cells and estimated cellularity (normalized to the size of tumor mass with necrosis) after cisplatin treatment in scramble and VIRMA knockdown conditions. * *p* < 0.05

Importantly, VIRMA-knockdown tumors showed significantly lower cell viability after cisplatin treatment than scramble tumors exposed to the same concentration of the drug. Histological representations of residual tumor after cisplatin treatment are presented in Fig. 6.

## Discussion

RNA modifications and related players involved in their regulation have been increasingly recognized in the past years as critical coordinators of several biological processes, both physiological and pathological, including cancer development [32–34]. m^6^A is the most abundant of these modifications in eukaryotic cells, which can be introduced in mRNA [and also, importantly, in other kinds of RNAs, like non-coding RNAs [35]] by different families of enzymes. They include the writers, which are assembled to form a methyltransferase complex (formed by METLL3, the component with catalytic activity, and other co-factors that recruit the complex, like VIRMA, WTAP and METTL14); the erasers (which remove the m^6^A modification, including ALKBH5 and FTO); and the readers (like the YTHDF-family of proteins, which recognize m^6^A and target the RNA molecule for a specific fate – translation, degradation or other - in a context-dependent manner) [36, 37]. This versatile level of expression regulation [so-called epitranscriptomics [38]] constitutes a quite recent but rapidly evolving field in cancer research [39, 40]. Indeed, these players have been implicated in several tumor models and in most stages of their development, from tumor initiation, to proliferation, invasion, metastatic dissemination, response/resistance to therapy and even modulation of the tumor microenvironment and phenomena like differentiation and epithelial-to-mesenchymal transition (EMT) [4, 41–45]. Few studies on TGCTs are available, though [10–13]. We hypothesize that owing to the intricate link of these tumors to embryonic and germ cell development [in which m^6^A is widely described as fundamental [18–20, 46–50]], investigation of such alterations holds the promise of identifying putative clinically useful biomarkers of the disease [51] and novel therapeutic approaches for these patients [52, 53]. Actually, this is supported by our in silico analysis of The Cancer Genome Atlas (TCGA) database [3], disclosing frequent dysregulation of these players in TGCTs in higher levels compared to other urological malignancies, namely of the writer VIRMA and reader YTHDF3, which we further validated in our tissue pilot study (including primary TGCTs and metastatic cisplatin-exposed samples) to be differentially expressed in these tumors according to histology and differentiation [10].

Herein, we first assessed the differential expression of several m^6^A-related players among cell lines representative of GCTs (three reflecting tumors of testicular and one of mediastinal origin). We observed some heterogeneity in expression of the different regulators: among the writers, the ones expressed at overall higher levels were WTAP, METTL3 and VIRMA, compared to METTL14 or METTL4. YTHDF3 was also expressed at high levels. Among the erasers, ALKBH5 was hardly expressed, while FTO expression was generally higher. These results suggest that WTAP, METTL3, VIRMA, YTHDF3 and FTO are key regulators involved in (T) GCTs biology. Of relevance, higher VIRMA and YTHDF3 expression levels were observed in the seminoma-like TCam-2 cells compared to the analyzed NS cell lines, in line with our previous observations in primary tissues in silico analysis [3, 10]. Despite showing higher mRNA expression of methyltransferase complex components, TCam-2 cells did not display the highest m^6^A abundance, which was also highly represented in embryonal carcinoma-related cells of NCCIT and NT2. We found, however, that m^6^A abundance in total RNA varied between 0.04 and 0.14% in TGCT cell lines, similar to the range reported by Nettersheim et al. [11].

TGCTs display distinctive degree of differentiation. The most differentiated form of TGCT, the TE, constitutes a clinical challenge; it is the single subtype not detectable by the most remarkable non-invasive biomarker of the disease, the miR-371a-3p, and classical serum markers show limited clinical usefulness in identifying TE [54]. Also, importantly, this is the most frequent subtype in post-chemotherapy metastatic specimens, constituting residual disease only potentially removed by surgical excision, which has associated risks. A better understanding of the biology of this subtype and of the differentiation process is needed to find new biomarkers and therapeutic targets. Since all (T) GCT cell lines display high expression of pluripotency factors [such as NANOG, OCT3/4 and LIN28 [55]] we decided to investigate the net effect in m^6^A methylome and respective writers, readers and erasers upon differentiation. For this, we differentiated NS cell lines using ATRA, as described before [24]. Remarkably, a statistically significant decrease of expression of most writers and of the reader YTHDF3 in differentiated cells was observed. This corroborates the significant decrease in m^6^A deposit in differentiated cells; being more impressive in NT2 cell line, the one in which the decrease in writers’ expression was also accompanied by a significant increase in expression of both erasers, FTO and ALKBH5. These data further reinforce the role of m^6^A in cellular fate decisions [46], also illustrated in this tumor model.

VIRMA has an oncogenic role in other tumor models [56–60], including urological malignancies [29]. Indeed, our previous observations highlighted frequent VIRMA upregulation in primary TGCTs (which was also confirmed in an independent study analyzing multiple cancer types [22]), with strong VIRMA immunoexpression found in 72.4% of samples and, in particular, in 84.2% of metastatic patient samples [3, 10]. For those reasons, we aimed at a better understanding of the biological implications in TGCTs. Our data corroborates the oncogenic properties of VIRMA, with its knockdown resulting in reduced tumor cell growth and decreased cell proliferation, as well as decreased cell migration and invasion. Additionally, this was further demonstrated in vivo, with reduced tumor size and angiogenesis, evaluated both macroscopically and histologically. We again highlight the versatility of the CAM assay for assessing tumor properties and response to drugs in vivo [61], also recently demonstrated for GCTs as well [62, 63]. Interestingly, VIRMA knockdown resulted in significant protein expression decrease of all members of the methyltransferase complex (METTL3, the only component with catalytic activity; METTL14, which is relevant for substrate recognition, specificity and activity; and WTAP, which stabilizes the complex). Our data supports the relevance of the regulatory function of VIRMA within the complex, that when impaired leads to decreased expression and less efficiency of the whole complex, as demonstrated by the statistically significant decrease in m^6^A deposition. Importantly, the assessment of m^6^A deposition was quantified (in addition to semi-quantitative dot blot), by a colorimetric ELISA-based method (used in several recent publications [64–66]), which includes pre-defined negative and positive controls, allowing for mathematical calculation of percentage of m^6^A with a sensitivity of 0.01 ng. This confirmed that changes observed in the VIRMA-knockdown cells occurred in a m^6^A-dependent manner. Indeed, VIRMA silencing was shown to decrease m^6^A deposit during embryonic development [67], constituting another link to developmental biology in this tumor model.

m^6^A-related proteins have also been implicated in therapy resistance in cancer [68]. Cisplatin resistance is a major clinical challenge in TGCTs, and targeted therapies are under investigation to treat these poor prognosis patients [69–72]. In our previous study we investigated 14 metastatic samples of patients exposed to cisplatin that developed resistance, and observed strong VIRMA immunoexpression in all 14 samples (illustrative examples of strong immunoexpression in cisplatin-resistant patient samples are represented in Supplementary Fig. 5, comparing to lower expression in cisplatin-sensitive primary TGCTs). In the current work we disclosed upregulation of the components of the methyltransferase complex in the cisplatin resistant clone of NCCIT cells (at mRNA and protein levels), suggesting that m^6^A dynamics is also indeed involved in the development of this resistant phenotype. Specifically, NCCIT resistant cells showed increased VIRMA mRNA levels (although not achieving statistical significance most likely due to limited number of replicates) but then confirmed by protein significant upregulation. This is in line with a recent study by Wei and collaborators [13], who demonstrated that METTL3 promotes resistance by introducing m^6^A in TFAP2C in the seminoma-like cell line TCam-2, both in vitro and in vivo. This indicates that METTL3 contributes to cisplatin resistance in the seminoma phenotype, and supports an oncogenic role for this player. There have been however some conflicting findings regarding the role of METTL3 in TGCTs, with another study [12] analyzing in silico data and showing METTL3 to be downregulated in TGCT tissues and lower expression to confer inferior disease-free survival (suggestive of a tumor suppressor role). Nonetheless, the same authors found increased proliferation, migration and invasion (by regulating EMT genes) in vitro, upon overexpression of this factor (suggestive of an oncogenic role). In our own patient cohort, METTL3 was highly expressed in tissue samples of primary and metastatic cisplatin pre-treated tumors (specific nuclear staining), with significantly higher immunoexpression in NS. However, and although higher expression occurred in patients with inferior disease-free survival, this did not reach statistical significance possibly owing to the limited sample number. This demonstrates that further investigation is needed to clarify the role of METTL3 in TGCT patients. For this, and based on our previous observations, we have instead focused on VIRMA for our in vitro investigations.

Although Wei and co-workers [13] have focused on cisplatin sensitive and resistant clones of the seminoma-like TCam-2 cell line, cisplatin resistance in the clinic is much more frequent in NS. We hence decided to look into the effect of VIRMA in cisplatin resistance emergence in the NS NCCIT cell line (the one showing the highest IC_50_ to cisplatin in our previous study [26]). Remarkably, we found that VIRMA knockdown cells were re-sensitized to cisplatin exposure, a finding confirmed in vivo, with VIRMA knockdown tumors showing significantly less viability after exposure to cisplatin compared to controls; thus, implicating a role of VIRMA in the acquisition of cisplatin resistance. Since the observed effect was not due to cell differentiation (as we found no significant difference in expression of pluripotency factors), we hypothesize that this was due to differential activation and regulation of DNA damage response, specifically of pathways involved in repair of double strand breaks elicited by cisplatin [69]. Interestingly, our results indicate that VIRMA knockdown cells sustained differentially more DNA damage, as demonstrated by increased expression of γH2AX and GADD45A/B. Moreover, VIRMA knockdown cells showed a more remarkable decrease in activation of several players involved in DNA repair, namely of XLF, involved in the non-homologous end joining DNA repair pathway. This pathway is involved in recognizing the breaks, recruiting repair complexes and processing/ligating the breaks [73, 74]. This is consistent with previous studies that implicated this DNA repair pathway and its players in cisplatin resistance (and to other agents, such as temozolomide in brain glioblastoma) in various tumor models, including hepatocellular, esophageal, breast and ovarian carcinomas [75–79], as well as recently reported in TGCTs [80]. Importantly, we found that both MRE11 and XLF showed several m^6^A sites predicted with high confidence (including a site with very high confidence for XLF transcript, which was the most differentially expressed DNA repair target). We believe that our experimental approach (1. CRISPR/Cas9-mediated knockdown of VIRMA, with confirmed decreased expression of the remaining methyltransferase complex and reduced m^6^A deposition; 2. the normalization to scramble vector to control for off-target effects in all experiments; and 3. the additional normalization to cisplatin exposure – versus no exposure/control) combined with the high score prediction of m^6^A sites in these transcripts endorses that changes occurred in a m^6^A-dependent manner, which were influenced by action of VIRMA within the complex.

The study of RNA modifications in cancer is recent but is growing fast, with multiple studies in the last 2 years attempting to understand the biological mechanisms of m^6^A gene expression regulation. The ultimate aim is, however, to explore the epitranscriptome and its players as therapeutic targets [81]. Although few inhibitors have been recently investigated in in vitro / in vivo settings [81] (most inhibiting the eraser FTO [82]), and despite a very recent and remarkable report of a METTL3 inhibitor with anti-cancer properties documented in myeloid leukemia [83], most inhibitors are rather non-selective and have limited target potency. Indeed, therapeutic targeting of these players is still in its embryonal stage and no clinical trials are currently available for agents targeting m^6^A writers, nor data reporting specific inhibitors of VIRMA [84], thus abrogating a therapeutic read-out from our study. However, this soon will be surpassed [81], as more researchers are now aware of the promise of targeting these players. Indeed, epitranscriptome pharmacologically targeting is seen with enthusiasm and most likely future specific inhibitors will be designed and assessed for their clinical efficacy [85]. We believe our data further endorses this evolving area of research, including in TGCTs, suggesting that they may be effective in the event of resistance to standard chemotherapy [52, 53, 81].

## Conclusions

Overall, we demonstrate that shifts in m^6^A abundance, as well as in expression of related players go along with the process of differentiation in (T) GCTs, and further highlight that VIRMA has an oncogenic role in these tumors, contributing both to tumor aggressiveness and to cisplatin response in NS, in vitro and in vivo, by influencing DNA repair capacity. In future studies we aim to further explore other pathways hypothetically regulated by m^6^A deposition, and evaluate DNA damage response in more detail, following our previous observations in this tumor model [86]. Our data further reinforces investigation of RNA modifications in TGCTs (with VIRMA representing a promising predictive biomarker of patient outcome and therapeutic target, to be confirmed in future studies). Importantly, future investigations of how interfering with m^6^A levels and its players may affect DNA repair proficiency may be instrumental to better understand cisplatin resistance in this and other tumor models, such as urothelial, esophageal, head and neck and lung cancers.

## Supplementary Information

**Additional file 1: Supplementary Fig. 1.** Differential abundance of m^6^A and expression of m^6^A related players upon ATRA-induced differentiation of non-seminoma cell lines. Differential mRNA expression levels of METTL3, WTAP, VIRMA, METTL14, METTL4, YTHDF3, ALKBH5 and FTO in differentiated NCCIT (A), NT2 (B) and 2102Ep (C), expressed as fold-change compared to control condition. Results are normalized to GUSB/18S rRNA, and plotted in 2^^-ΔΔCt^ format; D – Differential m^6^A abundance in NCCIT, NT2 and 2102Ep cells differentiated with ATRA, expressed as fold-change compared to control condition. * *p* < 0.05; ** *p* < 0.01.

**Additional file 2: Supplementary Fig. 2.** METTL3 immunoexpression in patient tumor samples. METTL3 immunoexpression in seminomas compared to non-seminomas (A), among the various TGCT individual subtypes (B), in TGCT cisplatin exposed metastatic tumors (C) and in relation to tumor stage (D); Disease-free survival of patients in respect to METTL3 immunoexpression. Results are computed using combined score of intensity and percentage of positive cells (see methods). **** *p* < 0.0001.

**Additional file 3: Supplementary Fig. 3.** Illustrative examples of METTL3 immunoexpression in TGCT patient samples. A – High immunoexpression score in a choriocarcinoma; B – High immunoexpression score in a yolk sac tumor; C – High immunoexpression score in a mixed tumor composed of embryonal carcinoma and yolk sac tumor; D – High immunoexpression score in a teratoma; E – Low immunoexpression score in a seminoma; F – Low immunoexpression score in a seminoma (left) but high immunoexpression score in another seminoma (right). All photomicrographs were taken in 400x magnification.

**Additional file 4: Supplementary Fig. 4.** Differential mRNA expression of pluripotency factors in VIRMA knockdown cells compared to scramble condition. Results are normalized to GUSB, computed in 2^^-ΔΔCt^ format and expressed as fold-change compared to scramble condition. n.s. – non significant.

**Additional file 5: Supplementary Fig. 5.** Illustrative examples of VIRMA immunoexpression in cisplatin-sensitive and -resistant patient samples. A and B – Embryonal carcinoma metastatic to lung in a cisplatin resistant patient. The patient was treated with multiple courses of platin-based therapy but showed disease progression, and died from disease; C – Mixed tumor composed of yolk sac tumor and teratoma metastatic to the brain in a cisplatin-resistant patient (*left*) and the corresponding primary testicular tumor (also a mixed tumor, composed of embryonal carcinoma, yolk sac tumor and teratoma, *right*) both showing strong nuclear VIRMA immunoexpression; D-F – Three primary TGCTs (a pure seminoma and two mixed tumors composed of embryonal carcinoma, teratoma and yolk sac tumor, respectively), chemo-naïve, with weak/moderate VIRMA immunoexpression. Patients received adjuvant platin-based chemotherapy and were free of disease, showing no relapses.

**Additional file 6: Supplementary Table 1.** Primer sequences used in the work.

**Additional file 7: Supplementary Table 2.** Antibodies used in the work.

**Additional file 8: Supplementary Table 3.** Clinicopathological features of the study cohort.

**Additional file 9: Supplementary Table 4.** SRAMP analysis of XLF for m6A predicted sites.

## Data Availability

All data generated or analyzed during this study are included in this published article and its supplementary information files.

## Abbreviations

ATRA: all-trans retinoic acid
BSA: bovine serum albumin
CAM: chorioallantoic membrane
CH: choriocarcinoma
CS: combined score
EC: embryonal carcinoma
EMT: epithelial to mesenchymal transition
GCNIS: germ cell neoplasia in situ
GCT: germ cell tumor
m^6^A: N6-methyladenosine
NS: non-seminoma
RT-qPCR: real-time quantitative polymerase chain reaction
SE: seminoma
TCGA: The Cancer Genome Atlas
TE: teratoma
TGCT: testicular germ cell tumor
YST: yolk sac tumor
