# Supplementary figures and images for: The component of the m6A writer complex VIRMA is implicated in aggressive tumor phenotype, DNA damage response and cisplatin resistance in germ cell tumors
Source: J Exp Clin Cancer Res. 2021 Aug 25;40:268. doi: 10.1186/s13046-021-02072-9 (PMC8390281; doi:10.1186/s13046-021-02072-9)

**A**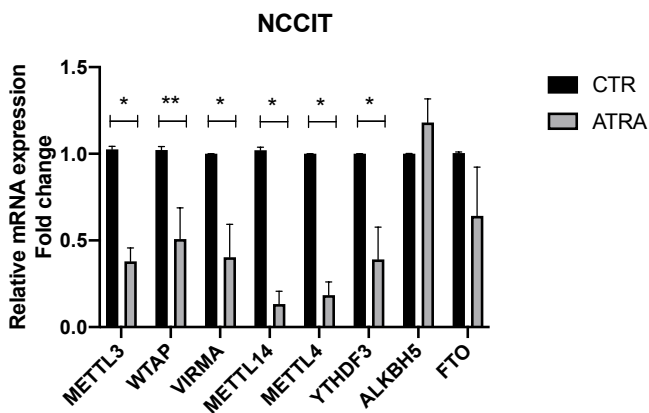**B**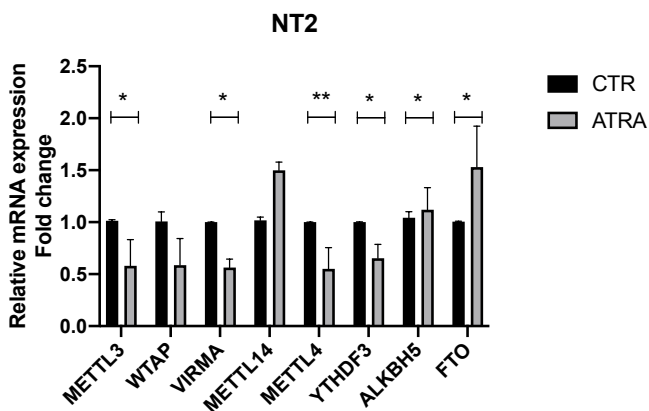**C**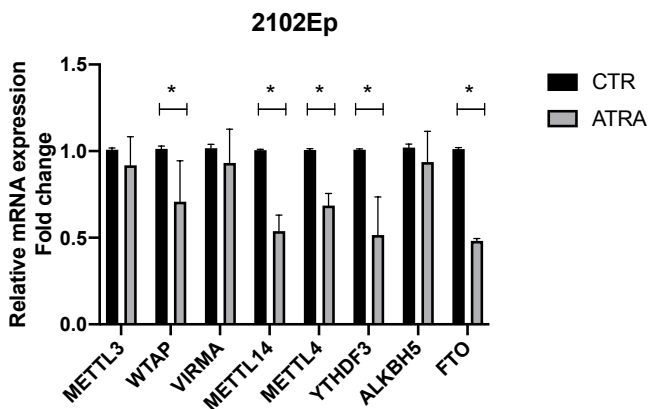**D**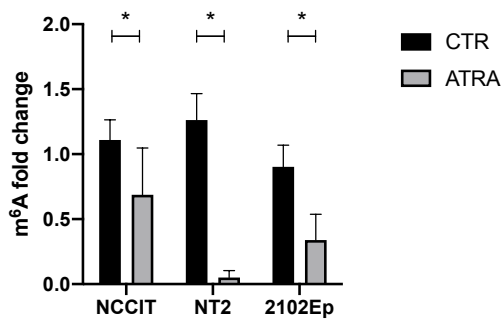

Supplement: Supplementary file 1 — Additional file 1: Supplementary Fig. 1. Differential abundance of m6A and expression of m6A related players upon ATRA-induced differentiation of non-seminoma cell lines. Differential mRNA expression levels of METTL3, WTAP, VIRMA, METTL14, METTL4, YTHDF3, ALKBH5 and FTO in differentiated NCCIT (A), NT2 (B) and 2102Ep (C), expressed as fold-change compared to control condition. Results are normalized to GUSB/18S rRNA, and plotted in 2^-ΔΔCt format; D – Differential m6A abundance in NCCIT, NT2 and 2102Ep cells differentiated with ATRA, expressed as fold-change compared to control condition. * p < 0.05; ** p < 0.01. [file 13046_2021_2072_MOESM1_ESM.pdf]

**A**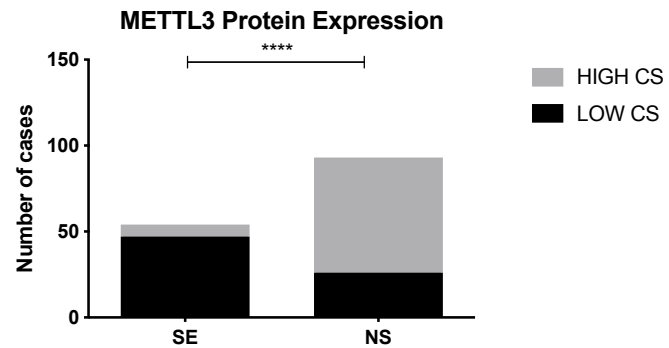**B**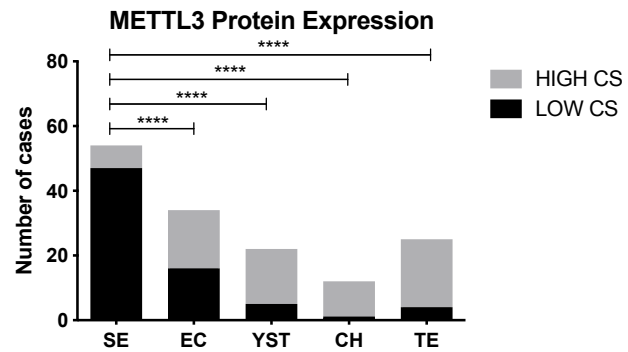**C**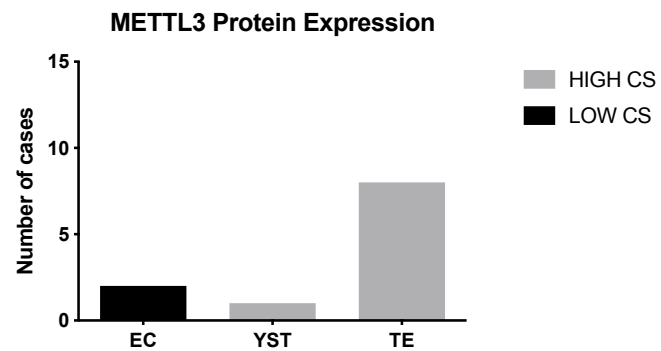**D**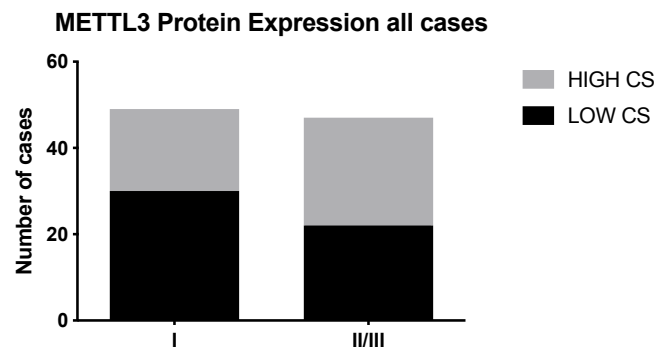**E**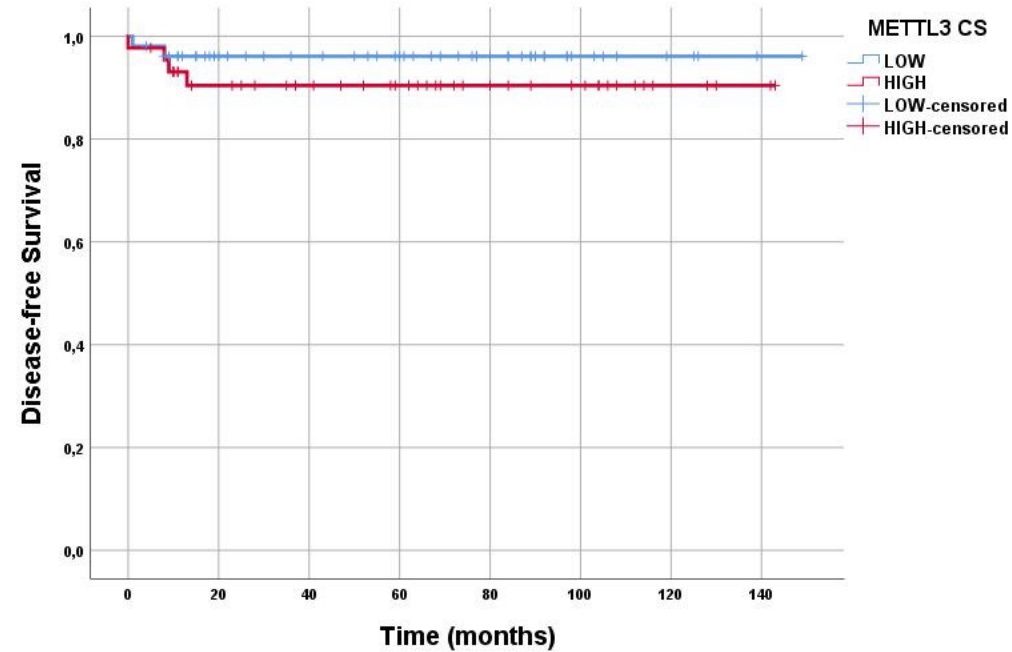

Supplement: Supplementary file 2 — Additional file 2: Supplementary Fig. 2. METTL3 immunoexpression in patient tumor samples. METTL3 immunoexpression in seminomas compared to non-seminomas (A), among the various TGCT individual subtypes (B), in TGCT cisplatin exposed metastatic tumors (C) and in relation to tumor stage (D); Disease-free survival of patients in respect to METTL3 immunoexpression. Results are computed using combined score of intensity and percentage of positive cells (see methods). **** p < 0.0001. [file 13046_2021_2072_MOESM2_ESM.pdf]

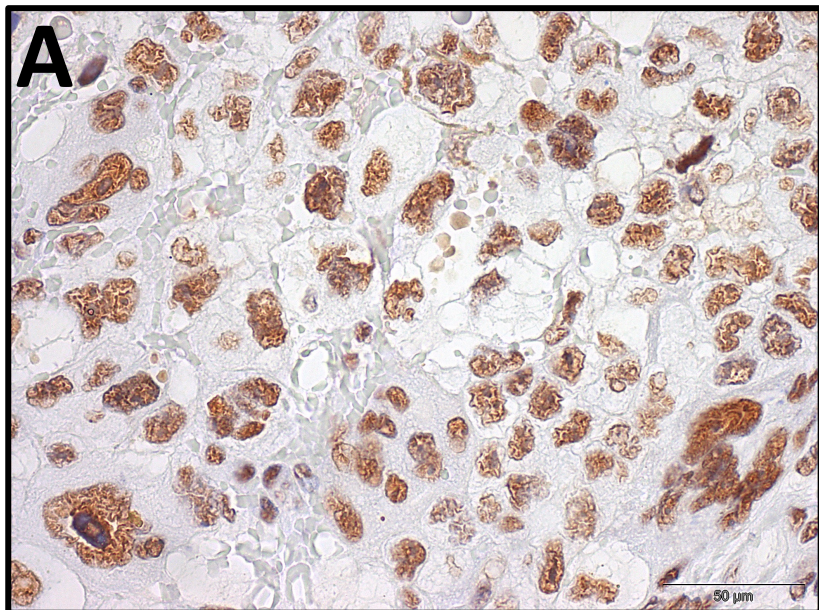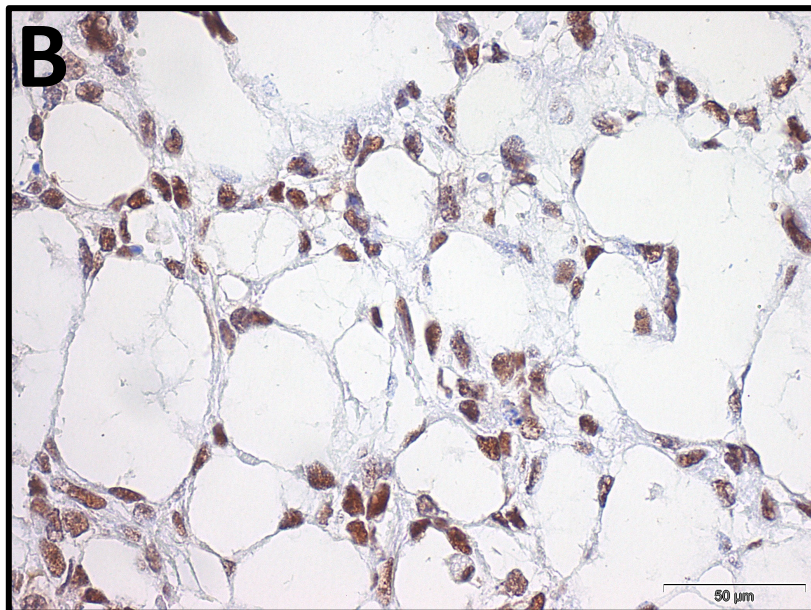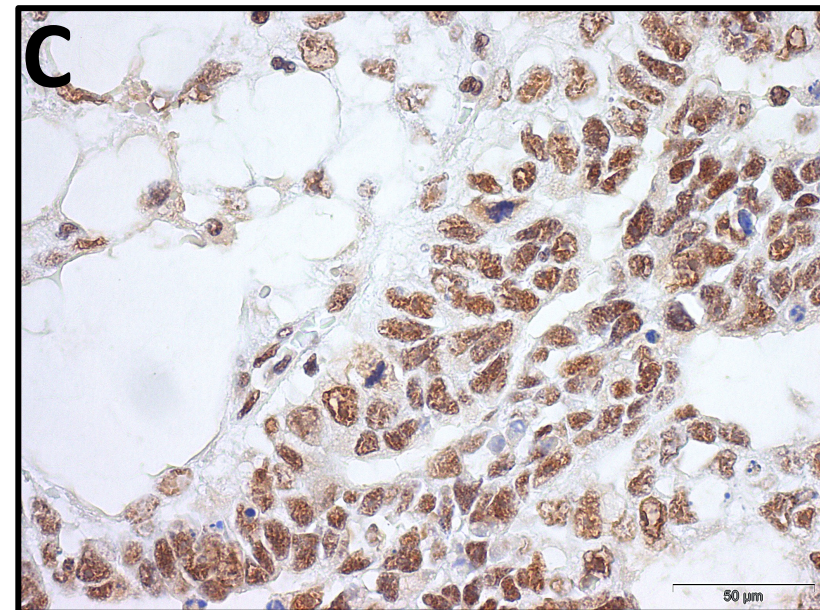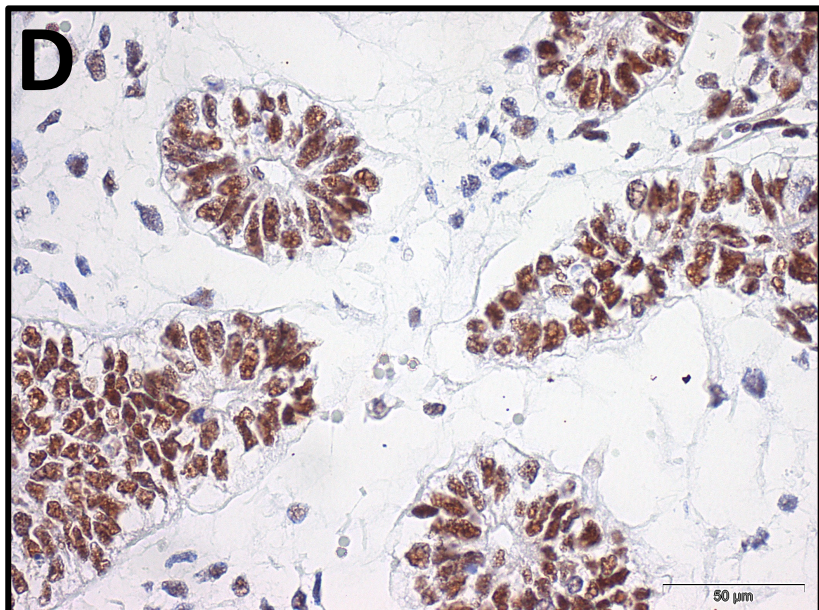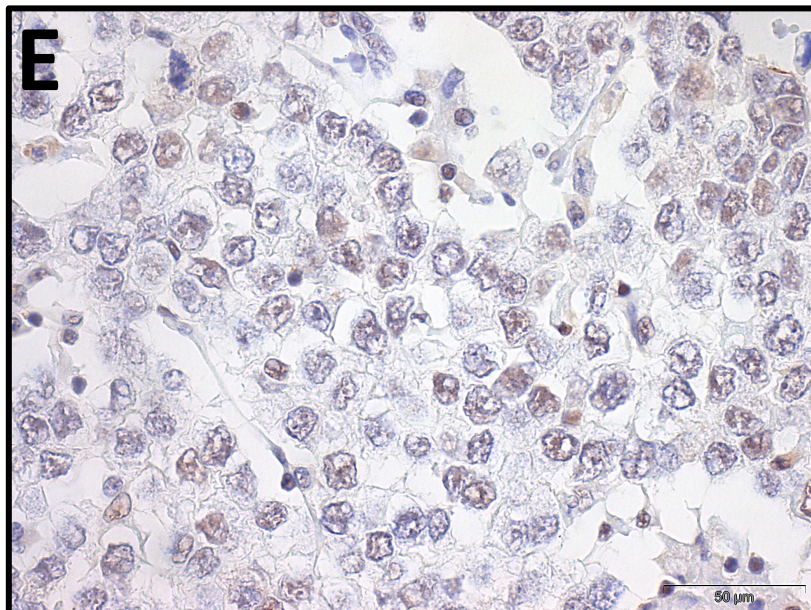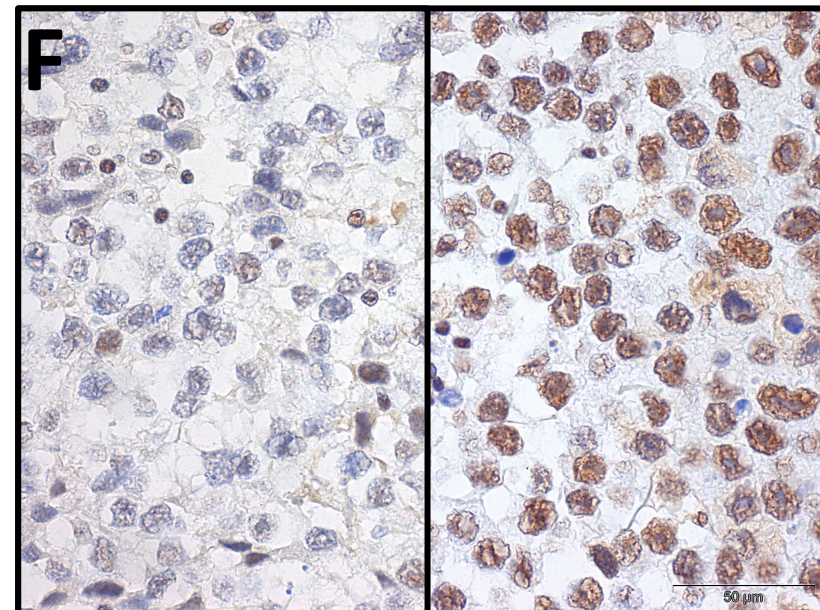

Supplement: Supplementary file 3 — Additional file 3: Supplementary Fig. 3. Illustrative examples of METTL3 immunoexpression in TGCT patient samples. A – High immunoexpression score in a choriocarcinoma; B – High immunoexpression score in a yolk sac tumor; C – High immunoexpression score in a mixed tumor composed of embryonal carcinoma and yolk sac tumor; D – High immunoexpression score in a teratoma; E – Low immunoexpression score in a seminoma; F – Low immunoexpression score in a seminoma (left) but high immunoexpression score in another seminoma (right). All photomicrographs were taken in 400x magnification. [file 13046_2021_2072_MOESM3_ESM.pdf]

# NCCIT

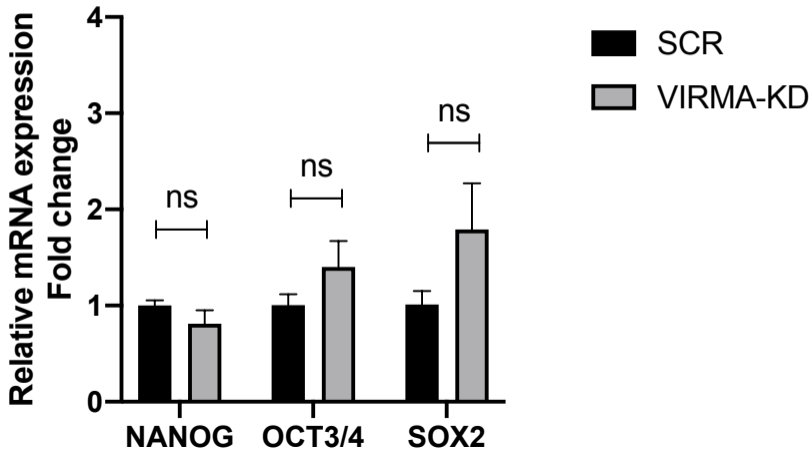

Supplement: Supplementary file 4 — Additional file 4: Supplementary Fig. 4. Differential mRNA expression of pluripotency factors in VIRMA knockdown cells compared to scramble condition. Results are normalized to GUSB, computed in 2^-ΔΔCt format and expressed as fold-change compared to scramble condition. n.s. – non significant. [file 13046_2021_2072_MOESM4_ESM.pdf]

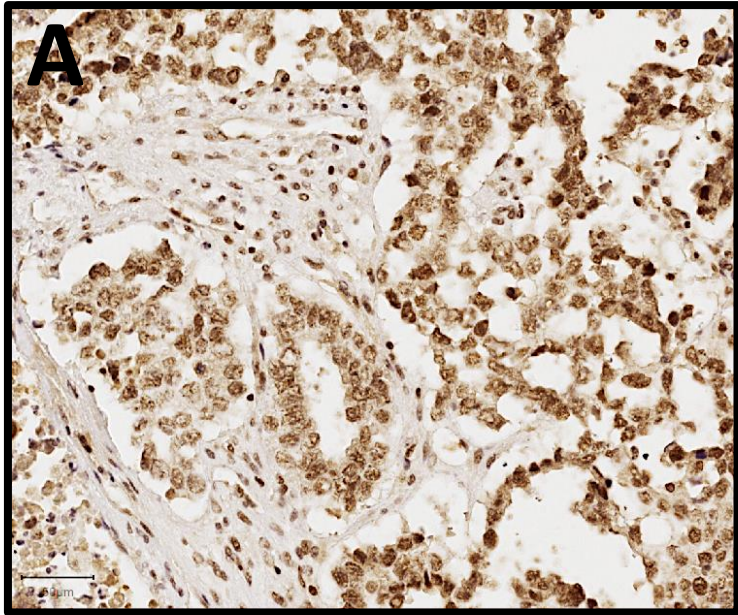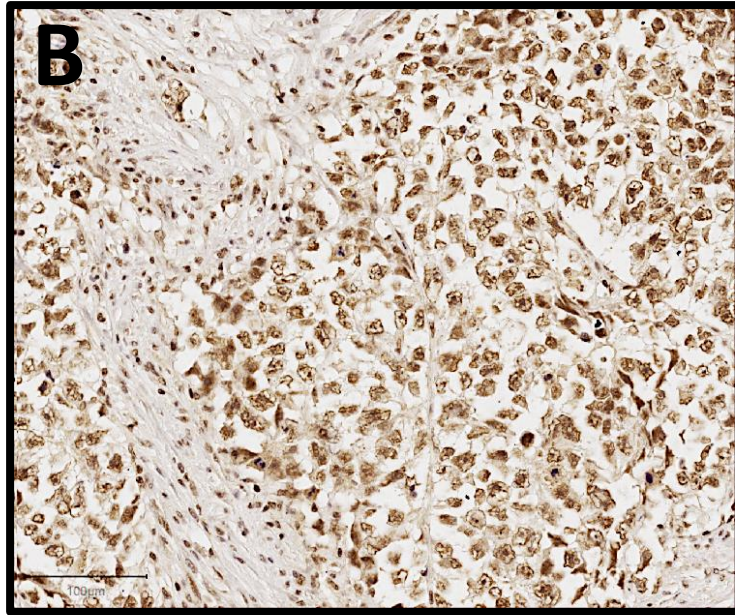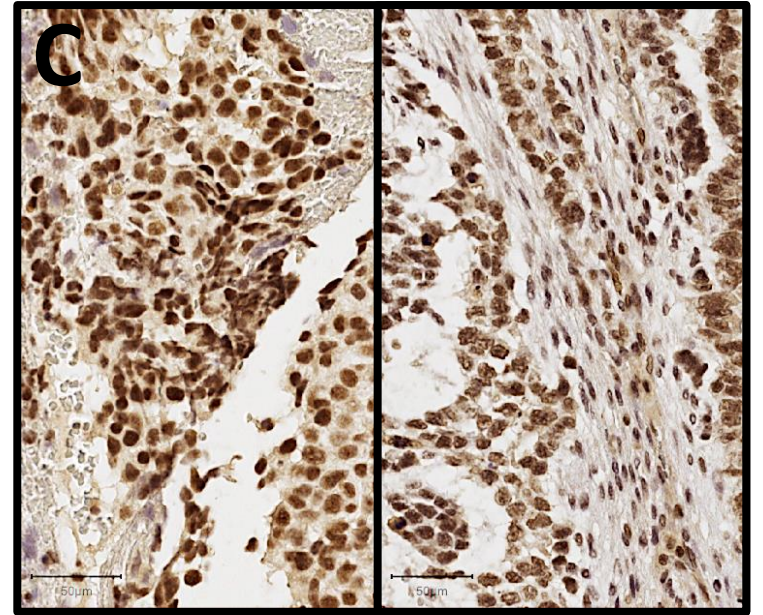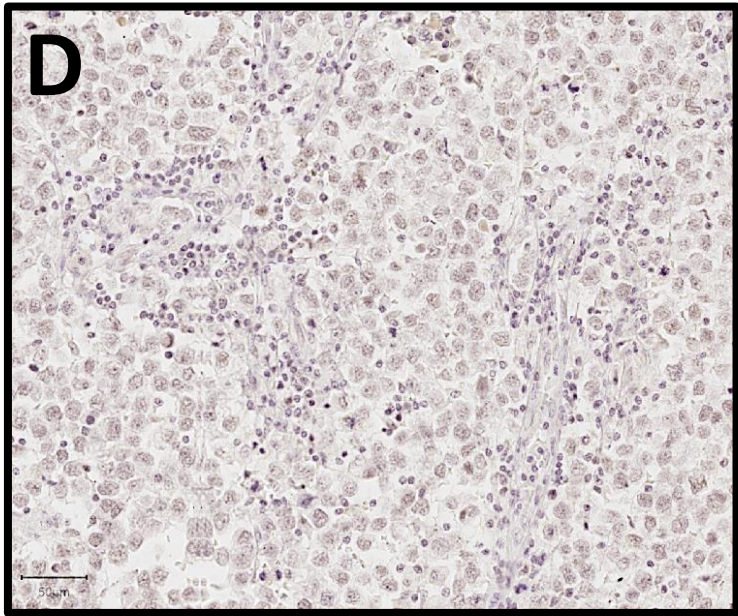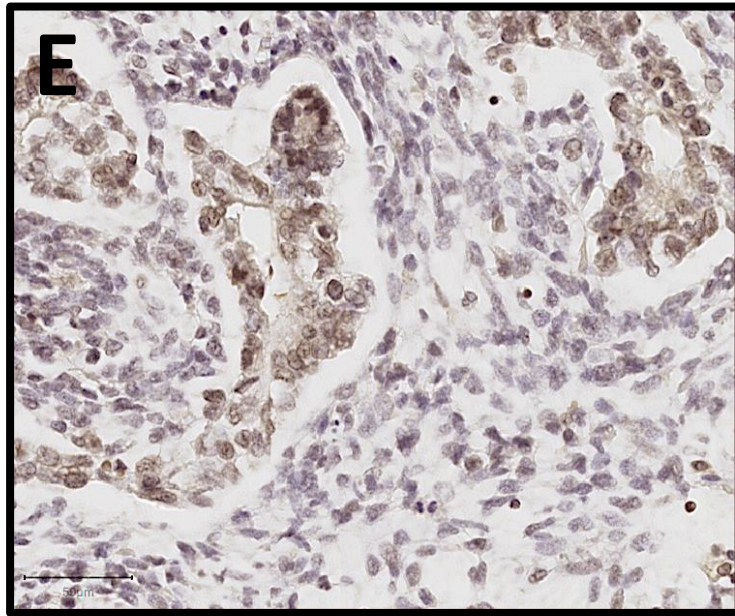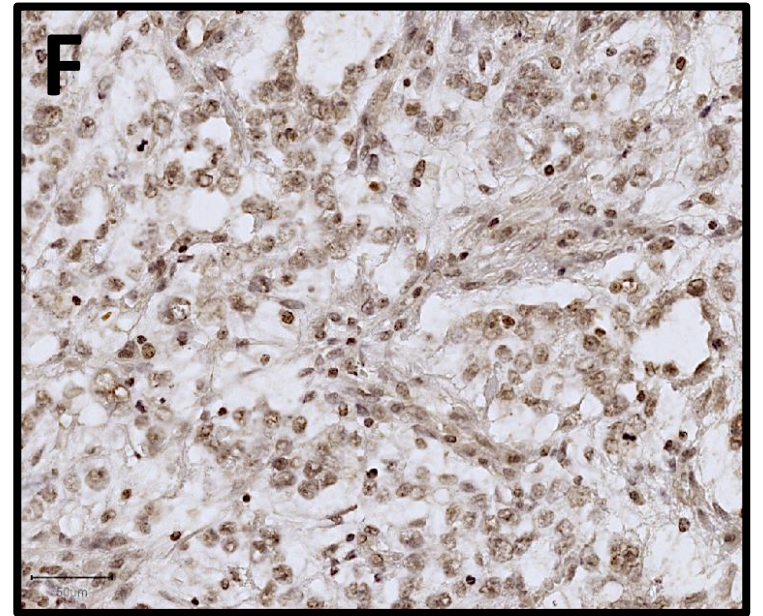

Supplement: Supplementary file 5 — Additional file 5: Supplementary Fig. 5. Illustrative examples of VIRMA immunoexpression in cisplatin-sensitive and -resistant patient samples. A and B – Embryonal carcinoma metastatic to lung in a cisplatin resistant patient. The patient was treated with multiple courses of platin-based therapy but showed disease progression, and died from disease; C – Mixed tumor composed of yolk sac tumor and teratoma metastatic to the brain in a cisplatin-resistant patient (left) and the corresponding primary testicular tumor (also a mixed tumor, composed of embryonal carcinoma, yolk sac tumor and teratoma, right) both showing strong nuclear VIRMA immunoexpression; D-F – Three primary TGCTs (a pure seminoma and two mixed tumors composed of embryonal carcinoma, teratoma and yolk sac tumor, respectively), chemo-naïve, with weak/moderate VIRMA immunoexpression. Patients received adjuvant platin-based chemotherapy and were free of disease, showing no relapses. [file 13046_2021_2072_MOESM5_ESM.pdf]
