# Supplementary material for: The component of the m6A writer complex VIRMA is implicated in aggressive tumor phenotype, DNA damage response and cisplatin resistance in germ cell tumors
Source: J Exp Clin Cancer Res. 2021 Aug 25;40:268. doi: 10.1186/s13046-021-02072-9 (PMC8390281; doi:10.1186/s13046-021-02072-9)
Supplement: Supplementary file 6 — Additional file 6: Supplementary Table 1. Primer sequences used in the work. [file 13046_2021_2072_MOESM6_ESM.docx]

**Supplementary Table 1: Primer sequences used in the work.**

| Gene | Primer sequence  (5’-3’) | 10μM primers, volume  (μL), F+R | Annealing  Temperature  (°C) |
| --- | --- | --- | --- |
| NANOG | F: TGAACCTCAGCTACAAACAGGTG | 0.5 | 60 |
|  | R: AACTGCATGCAGGACTGCAGAG |  |  |
| OCT3/4 | F: CTTGCTGCAGAAGTGGGTGGAGGAA | 0.4 | 64 |
|  | R: CTGCAGTGTGGGTTTCGGGCA |  |  |
| SOX2 | F: AGAACCCCAAGATGCACAAC | 0.5 | 60 |
|  | R: CGGGGCCGGTATTTATAATC |  |  |
| PAX6 | F: AACGATAACATACCAAGCGTGT | 0.5 | 62 |
|  | R: GGTCTGCCCGTTCAACATC |  |  |
| METTL3 | F: CAAGCTGCACTTCAGACGAA | 0.5 | 60 |
|  | R: GCTTGGCGTGTGGTCTTT |  |  |
| FTO | F: GAAAATCTGCTGGACAGCTCA | 0.5 | 60 |
|  | R: CGAGAGAGTCATCCTCACTT |  |  |
| YTHDF3 | Gene expression assay | N/A | 60 |
| VIRMA | Gene expression assay | N/A | 60 |
| ALKBH5 | Gene expression assay | N/A | 60 |
| WTAP | F: TTCCCAAGAAGGTTCGATTG | 0.5 | 60 |
|  | R: TGCAGACTCCTGCTGTTGTT |  |  |
| METTL14 | F: AGAAACTTGCAGGGCTTCCT | 0.5 | 60 |
|  | R: TCTTCTTCATATGGCAAATTTTCTT |  |  |
| RAD9 | F: GTGAAGGTGCTCGGCAAGG | 0.5 | 60 |
|  | R: CCAAGGGTTCCAGGTAGAGC |  |  |
| GADD45A | F: AGCAGAAGACCGAAAGGATGG | 0.3 | 60 |
|  | R: TGACTCAGGGCTTTGCTGAG |  |  |
| GADD45B | F: CTGGTCACGAACCCTCACAC | 0.5 | 60 |
|  | R: CTTTCTTCGCAGTAGCTGGC |  |  |
| METTL4 | Gene expression assay | N/A | 60 |
| 18SrRNA | Gene expression assay | N/A | 60 |
| GUSB | F: ATGCCATCGTGTGGGTGAAT | 0.5 | 60 |
|  | R: TGGACCAGGTTGCTGATGTC |  |  |
