# Supplementary material for: The component of the m6A writer complex VIRMA is implicated in aggressive tumor phenotype, DNA damage response and cisplatin resistance in germ cell tumors
Source: J Exp Clin Cancer Res. 2021 Aug 25;40:268. doi: 10.1186/s13046-021-02072-9 (PMC8390281; doi:10.1186/s13046-021-02072-9)
Supplement: Supplementary file 7 — Additional file 7: Supplementary Table 2. Antibodies used in the work. [file 13046_2021_2072_MOESM7_ESM.docx]

**Supplementary Table 2 – Antibodies used in the work**

| **Antibodies** | **Vendor** | **Catalog number** | **Dilution** |
| --- | --- | --- | --- |
| Anti-METTL3 | Abcam | ab195352 | 1/1000 1h at RT (1:750 for immunohistochemistry) |
|  |  |  |  |
| Anti- METTL14 | Abcam | ab220031 | 1/1000 1h at RT |
|  |  |  |  |
| Anti-WTAP | Abcam | ab195380 | 1/1000 1h at RT |
|  |  |  |  |
| Anti-VIRMA/Virilizer | Cell Signaling | 88358 | 1/1000 overnight at 4^o^C |
|  |  |  |  |
|  |  |  |  |
|  |  |  |  |
| Phospho Histone γH2AX (Ser139) | Cell Signaling | 9718 | 1/500 (immunofluorescence) |
| Anti-XLF | Cell Signaling | 2854T | 1/1000 overnight at 4ºC |
| Anti-MRE11 | Cell Signaling | 4847T | 1/1000 overnight at 4ºC |
| Anti-KU80 | Cell Signaling | 2180T | 1/1000 overnight at 4ºC |
| Anti-DNA-PKcs | Cell Signaling | 4602s | 1/1000 overnight at 4ºC |
| Anti-RAD50 | Cell Signaling | 3427T | 1/1000 overnight at 4ºC |
| Anti-NSB1 | Cell Signaling | 3001T (Ser343) | 1/1000 overnight at 4ºC |
| Anti-ATM | Cell signaling | 2873T | 1/500 overnight at 4ºC |
| Anti-β-Actin | Sigma-Aldrich | A1978 | 1/10 000 1h at RT |
|  |  |  |  |
