# Supplementary material for: The component of the m6A writer complex VIRMA is implicated in aggressive tumor phenotype, DNA damage response and cisplatin resistance in germ cell tumors
Source: J Exp Clin Cancer Res. 2021 Aug 25;40:268. doi: 10.1186/s13046-021-02072-9 (PMC8390281; doi:10.1186/s13046-021-02072-9)
Supplement: Supplementary file 8 — Additional file 8: Supplementary Table 3. Clinicopathological features of the study cohort. [file 13046_2021_2072_MOESM8_ESM.docx]

**Supplementary Table 3. Clinicopathological features of the study cohort.**

| Variables | Primary TGCT cases (n, %) |
| --- | --- |
| Histologic subtypes – TGCT patients (n, %) |  |
| Pure seminoma | 48/96 (50.0) |
| Pure embryonal carcinoma | 11/96 (11.5) |
| Pure postpubertal-type teratoma | 2/96 (2.0) |
| Mixed tumor | 35/96 (36.5) |
| Histological subtypes – individual components (n, %) |  |
| Seminoma | 54/147 (36.7) |
| Embryonal carcinoma | 34/147 (23.1) |
| Postpubertal-type yolk sac tumor | 22/147 (15.0) |
| Choriocarcinoma | 12/147 (8.2) |
| Postpubertal-type teratoma | 25/147 (17.0) |
| Stage (n, %) |  |
| I | 49/96 (51.0) |
| II | 28/96 (29.2) |
| III | 19/96 (19.8) |

Abbreviations: TGCT – testicular germ cell tumors.
