# Supplementary material for: The component of the m6A writer complex VIRMA is implicated in aggressive tumor phenotype, DNA damage response and cisplatin resistance in germ cell tumors
Source: J Exp Clin Cancer Res. 2021 Aug 25;40:268. doi: 10.1186/s13046-021-02072-9 (PMC8390281; doi:10.1186/s13046-021-02072-9)
Supplement: Supplementary file 9 — Additional file 9: Supplementary Table 4. SRAMP analysis of XLF for m6A predicted sites. [file 13046_2021_2072_MOESM9_ESM.docx]

**Supplementary Table 4. SRAMP analysis of XLF and MRE11 for m^6^A predicted sites.**

| XLF | | | | |
| --- | --- | --- | --- | --- |
| # m^6^A sites | Position | Sequence context | Score (combined) | Decision |
| 1 | 1128 | GGCCT CCTGT AAGGG CGAGA GAACT GAAGT TGATG TTGAC AGGCC | 0.603 | m^6^A site (**Moderate** confidence) |
| 2 | 1144 | GAGAG AACTG AAGTT GATGT TGACA GGCCC ACAGG GAATT GGCCT | **0.904** | m^6^A site (**Very high** confidence) |
| 3 | 1255 | TAGGT TCAGG TTTCT ACCAT GGACT TTAGG TATAT AGGGC AAGTC | 0.603 | m^6^A site (**Moderate** confidence) |
| 4 | 1584 | TTTGG AAACT TTGAT CTCAA GAACT CTCTT GATGG TGGGC ACAAG | 0.559 | m^6^A site (**Low** confidence) |
| 5 | 2191 | TGGAT CCTGA AGTGA GTAAG TGACT TATCA AAGGT CATAC AGCTA | 0.566 | m^6^A site (**Low** confidence) |
| 6 | 2304 | AAACA TTTAT TTTCC AGAAA GGACA TTAGG TTCTT CTTTC CAGTC | 0.575 | m^6^A site (**Low** confidence) |
| 7 | 2426 | AGGAG CTCTT GAGGA CTCCA GGACT TACCC TCATG CTTTT TCCTG | 0.609 | m^6^A site (**Moderate** confidence) |
| 8 | 2552 | TAGAG TGTGC TGTGT GGTAG TGACT GTCAT CGAAG TGAAT TGGGA | **0.675** | m^6^A site (**High** confidence) |
| 9 | 2575 | CTGTC ATCGA AGTGA ATTGG GAACT ATATA AAAAC AGCCT ATCAG | 0.562 | m^6^A site (**Low** confidence) |
| 10 | 3060 | TCCCT GTACT GTAAC AGCAC AGACT CAAGA CATTT TTCAT CACAG | **0.647** | m^6^A site (**High** confidence) |
| 11 | 3653 | GGGCT GGCTG GTGGG AGAAG GGACT CTTTC AATTG CTTTG GAGTA | **0.626** | m^6^A site (**High** confidence) |
| 12 | 3738 | AAGCC AGCAA ACCAG GAGCA GGACT TGGGA CCTCG AGGAA GTTCC | **0.626** | m^6^A site (**High** confidence) |
| 13 | 3768 | CCTCG AGGAA GTTCC TGCAG GAACT TGACC TAAAA AGTAG AGAAA | 0.572 | m^6^A site (**Low** confidence) |
| 14 | 4182 | GATCT GTGCT TCTAT GCTCT GAACT GGAAA GAGTC TAGCC TGTTG | 0.587 | m^6^A site (**Moderate** confidence) |
| 15 | 4916 | TCTAA TTTTT CTTGA AGAAA AAACT GTTAG AAAGG AATAA GTGAC | 0.594 | m^6^A site (**Moderate** confidence) |
| 16 | 5654 | TGTGG GGAGC ACACA GCCAT TGACT CACCT GGGAA GATTG TGCTA | 0.615 | m^6^A site (**Moderate** confidence) |
| 17 | 6062 | CAGAG TCCTC CTAGC TTTAT GGACC CCTGA TAGGA TTGAC GGCCA | 0.567 | m^6^A site (**Low** confidence) |
| 18 | 6742 | TGTGC TAAAG GGGAA ATTTA AGACT GGGCT CCATG GCTTA CGCCT | 0.597 | m^6^A site (**Moderate** confidence) |
| MRE11 | | | | |
| # m^6^A sites | Position | Sequence context | Score (combined) | Decision |
| 1 | 147 | AACCT GGTCC CAGAG GAGCT TGACT GACCA TAAAA ATGAG TACTG | 0.564 | m^6^A site (**Low** confidence) |
| 2 | 584 | GCAGA TGCAC TTTGT GCCTT GGACA TTTTA AGTTG TGCTG GATTT | 0.589 | m^6^A site (**Moderate** confidence) |
| 3 | 782 | TTGAG ACCAA AGGAA GATGA GAACT CTTGG TTTAA CTTAT TTGTG | 0.584 | m^6^A site (**Moderate** confidence) |
| 4 | 1262 | CCTCT TGTAC GACTG CGAGT GGACT ATAGT GGAGG TTTTG AACCT | **0.654** | m^6^A site (**High** confidence) |
| 5 | 1979 | GAGAC TTCTA CCCGT AGCAG GAACT CAAAG ACTGC TGTGT CAGCA | 0.607 | m^6^A site (**Moderate** confidence) |
| 6 | 1987 | TACCC GTAGC AGGAA CTCAA AGACT GCTGT GTCAG CATCT AGAAA | **0.643** | m^6^A site (**High** confidence) |
| 7 | 3727 | GATCT GCCCA CATCT TTATT GAACT TGAAA TCTAA TATTT CTAGT | 0.578 | m^6^A site (**Low** confidence) |
| 8 | 4622 | CTTTA ATTCA GGTAT GTAAA AGACT TTTTT CCCAA ACTTT TAAAA | 0.601 | m^6^A site (**Moderate** confidence) |
